# *Negr1* deficiency alters glutamate signalling and kynurenine pathway in a mouse model of psychiatric disorders

**DOI:** 10.1038/s41598-026-35968-7

**Published:** 2026-01-16

**Authors:** Carolin Kuuskmäe, Kaie Mikheim, Narges Mohammadrahimi, Kalle Kilk, Maria Kaare, Mohan Jayaram, German Ilnitski, Este Leidmaa, Mari-Anne Philips, Eero Vasar

**Affiliations:** 1 Department of Physiology, Institute of Biomedicine and Translational Medicine, University of Tartu, Tartu, Estonia; 2 Department of Biochemistry, Institute of Biomedicine and Translational Medicine, University of Tartu, Tartu, Estonia

**Keywords:** Negr1, NMDA, MK-801, Kynurenine pathway, Behavioural tolerance, Diseases, Neuroscience

## Abstract

The *NEGR1* gene has been implicated in several psychiatric disorders, and increased NMDA receptor binding density has been demonstrated in vitro in hippocampal slices from *Negr1*-deficient mice. In this study, we expanded on these findings by investigating the behavioural response to NMDA receptor antagonism, expression of NMDA receptor subunits, and kynurenine pathway metabolites in a *Negr1*-deficient mouse model. Male and female wild-type and *Negr1*-deficient mice received daily injections of MK-801, a non-competitive NMDA receptor antagonist, until behavioural tolerance developed in the open field test (after 9 days in males and 5 days in females). In drug-naive animals, acute MK-801 administration (0.2 mg/kg) elicited a stronger motor response in *Negr1*-deficient males compared to wild-type controls. However, with repeated dosing, *Negr1*-deficient males exhibited a blunted behavioural response and attenuated progression of rapid behavioural tolerance during every-second-day MK-801 administration, suggesting altered receptor sensitivity. Gene expression analysis revealed sex- and brain region-specific changes in NMDA receptor subunit expression. Additionally, kynurenine pathway metabolites showed genotype- and sex-dependent alterations. These findings suggest that NEGR1 protein modulates NMDA receptor function and tryptophan metabolism in a sex-dependent manner, highlighting the importance of considering both genetic background and sex in models of glutamatergic dysfunction relevant to neuropsychiatric disorders.

**Supplementary Information:**

The online version contains supplementary material available at 10.1038/s41598-026-35968-7.

## Introduction

Psychiatric disorders such as anxiety, major depression, bipolar disorder, and schizophrenia affect around 800 million people worldwide, often impairing quality of life^1,2^. Genetic factors, including polymorphisms in specific genes, contribute to their susceptibility. One such gene is neuronal growth regulator (*NEGR1)*. This gene encodes a cell adhesion molecule (NEGR1) involved in neural development, synapse formation, and plasticity^3–6^. Genome-wide association studies (GWAS) have identified *NEGR1* as a risk gene for several psychiatric and neurodevelopmental disorders^7–12^. However, the mechanisms through which NEGR1 influences behaviour and neurotransmitter systems remain poorly understood.

Recent evidence suggests that NEGR1 regulates synaptic function by modulating both inhibitory and excitatory signalling. NEGR1 promotes palmitoylation-dependent clearance of the GABA-synthesising enzyme GAD65 from the plasma membrane, thereby maintaining normal GABAergic synapse density and inhibitory tone. Loss of NEGR1 reduces GABAergic synapses and synaptic GABA levels, shifting the excitation–inhibition balance toward increased excitatory drive^13^. Moreover, our research group has previously shown that *Negr1*-deficient mice have a reduced number of parvalbumin-positive inhibitory interneurons in the hippocampus^5^. In parallel, NEGR1 has been implicated in glutamatergic AMPA receptor trafficking and dendritic spine maturation^14^. These processes are crucial for NMDA-dependent synaptic plasticity, suggesting that NEGR1 acts as a synaptic organiser coordinating GABAergic and glutamatergic communication. Its deficiency may thus disrupt the homeostatic regulation of excitatory neurotransmission relevant to psychiatric disorders. In the dentate gyrus of *Negr1*-deficient mice, long-term potentiation (LTP) and miniature excitatory postsynaptic current (mEPSC) frequency are markedly reduced^11^. These data indicate that NEGR1 could be required for balancing the ratio of excitation and inhibition in the brain.

Previous findings have shown that MK-801 (dizocilpine) binding density at NMDA receptors is higher in hippocampal sections of *Negr1*-deficient mice compared to wild-type (WT) controls, suggesting increased N-methyl-D-aspartate (NMDA) receptor availability in the *Negr1*-deficient brain^4^. Given the central role of NMDA receptors in synaptic plasticity, learning, and memory^15–17^, the glutamatergic system emerges as a potential pathway linking *Negr1* to psychiatric phenotypes. The NMDA receptor is composed of multiple subunits (e.g., GluN1, GluN2A, GluN2B), and changes in their expression have been associated with cognitive and emotional dysregulation^18,19^. Notably, GluN1 and GluN2A subunits serve as binding sites for D-serine, a molecule that can act as either a co-agonist or antagonist depending on the site^20^.

Our previous work demonstrated that *Negr1*-deficient mice exhibit heightened behavioural sensitivity to amphetamine, including exaggerated motor and stereotypic responses, along with altered expression of dopaminergic markers^8^. These findings suggest that *Negr1* influences dopaminergic reactivity and behavioural sensitisation. Building on prior in vitro findings of increased MK-801 binding to NMDA receptors in *Negr1*-deficient brain tissue, the present study investigates how MK-801, a non-competitive NMDA receptor antagonist known to mimic glutamatergic dysfunction and interfere with sensitisation processes^21–23^, affects behaviour and molecular markers of glutamate neurotransmission in *Negr1*-deficient mice.

We asked whether the expression of NMDA subunits or modulation by the NMDA co-agonist D-serine could be altered in *Negr1*-deficient mice. D-serine levels are regulated by serine racemase (Srr), an enzyme that is thus critical for NMDA receptor function. Disruptions in Srr activity have been implicated in schizophrenia spectrum disorders^24–26^. Dysregulation of NMDA receptor subunits and Srr activity may therefore provide a mechanistic link between *Negr1*-deficiency and the behavioural abnormalities observed in psychiatric conditions. In addition to direct glutamatergic modulation, we considered the role of the kynurenine pathway (KP), which metabolises tryptophan into neuroactive compounds such as kynurenic acid (KYNA) and quinolinic acid (QUIN). KYNA acts as an NMDA receptor antagonist at GluN1 subunits, while QUIN acts as an agonist at GluN2A and GluN2B subunits^19,27–31^. Imbalances in these metabolites have been associated with psychiatric and neurodegenerative disorders^19,32,33^, suggesting that KP dysregulation may influence NMDA receptor function and excitatory signalling.

Despite growing evidence implicating the KP in neuropsychiatric conditions, the relationships between *Negr1*, NMDA receptor signalling, KP metabolites, and glutamate levels remain poorly defined.

Thus, the present study aims to elucidate how *Negr1* deficiency affects behaviour and its underlying molecular mechanisms, focusing specifically on glutamatergic signalling and kynurenine pathway metabolism. Using a *Negr1-*deficient mouse model, we examined the expression of key NMDA receptor subunits (GluN1, GluN2A, GluN2B) and serine racemase (Srr) in the hippocampus and frontal cortex—regions crucial for learning, memory, and higher cognitive functions that depend on NMDA receptor-mediated plasticity^15,34,35^. Additionally, we measured kynurenine pathway metabolites and glutamate levels, both known modulators of NMDA receptor activity^34,35,36^ and implicated in neuropsychiatric disease^37–39^. To evaluate behavioural and molecular sensitivity to glutamatergic disruption, we assessed responses to repeated MK-801 administration. Finally, we investigated sex differences in these outcomes to determine whether *Negr1*-related effects differ between male and female mice. By linking behavioural phenotypes with glutamatergic and metabolic alterations, this study provides new insights into the neurobiological mechanisms underlying psychiatric disorders associated with NEGR1.

## Results

### Effect of repeated treatment with MK-801 (0.2 mg/kg) on locomotor activity in male wild-type and *Negr1*-deficient mice

**Fig. 1:**
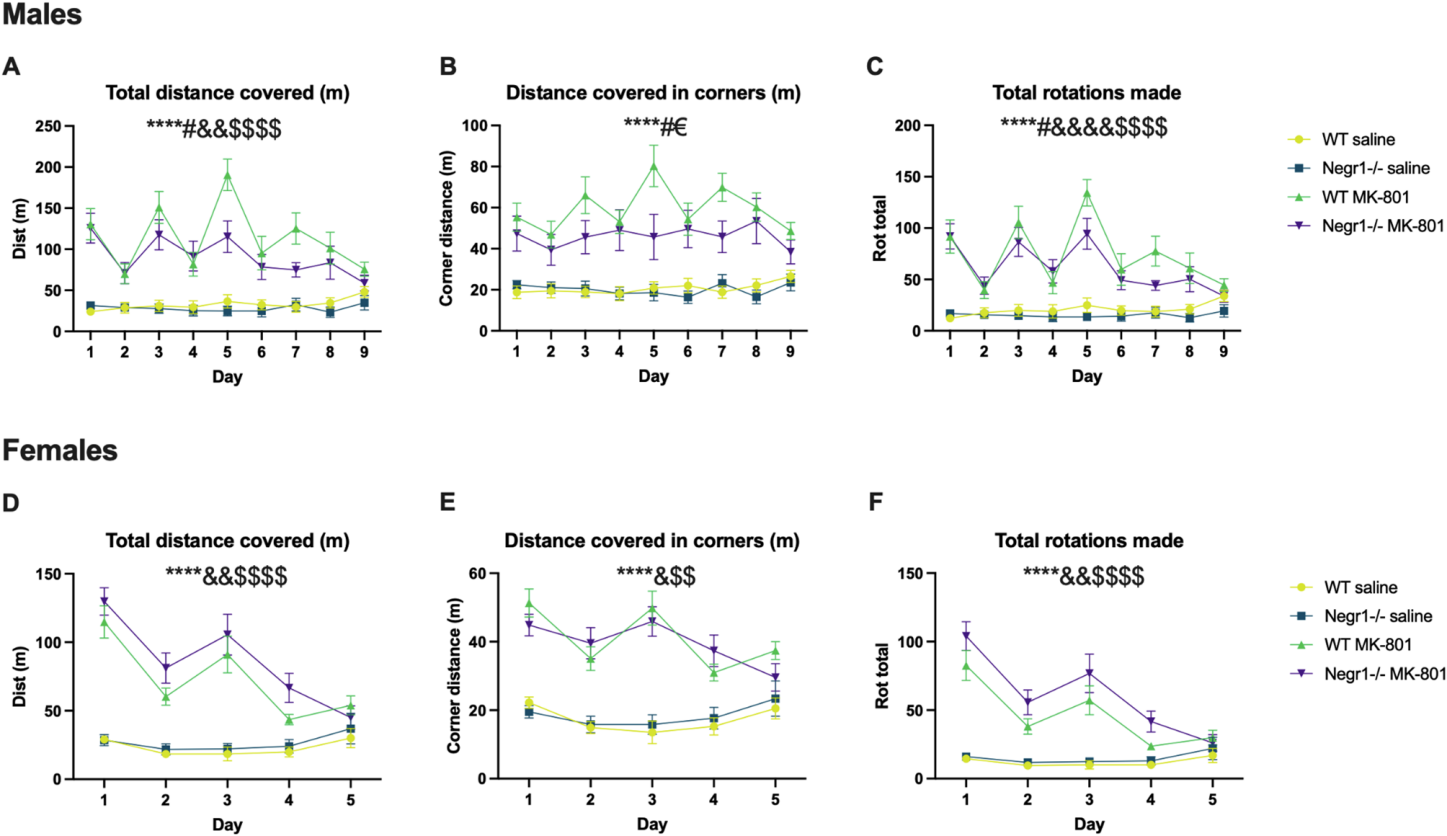
MK-801 effect on wild-type (WT) and *Negr1*-deficient mice’s behaviour in the open field test. Figure shows the total distance covered (**A**,** D**), distance covered in corners (**B**,** E**) and total rotations made (**C**,** F**) by both male and female mice until behavioural tolerance developed (after 9 days in males and 5 days in females). Each dot represents the day’s average (males *n* = 10, females *n* = 8–16), whiskers show SEM. Main effects, calculated using three-way ANOVA (Tukey HSD test), are depicted as symbols above graphs: * - treatment, # - genotype, & - day, $ - day and treatment interaction, € - genotype and treatment interaction. One symbol - *p* < 0.05, two symbols - *p* < 0.01, three symbols - *p* < 0.001, four symbols *p* < 0.0001. Exact values can be found in the Supplementary Table S1.

Based on the dose–response experiments performed in male mice, the optimal dose for behavioural activation was determined to be 0.2 mg/kg (Supplementary Fig. S1A-C). Acute administration of MK-801 at this dose produced a significantly stronger motor activity response in *Negr1*-deficient (*Negr1*^*−/−*^) mice compared to wild-type controls (total distance covered - *p* < 0.0001; distance covered in corners - *p* < 0.05). Interestingly, this enhanced response was not observed during the first day of testing in the repeated administration experiment (Fig. 1A-C). Notably, the same cohort of mice used for the dose-response curve, following a one-week washout period, was also used for the repeated administration protocol. As a result, these mice were not completely drug-naive at the start of the repeated treatment.

We hypothesised that the heightened acute response to MK-801 is specific to drug-naive *Negr1*-deficient mice. To test this, the acute administration experiment was repeated in an independent cohort of drug-naive male mice. Consistent with our hypothesis, *Negr1*-deficient mice in this new cohort again showed a stronger motor activity response, as measured by the total distance covered (*p* < 0.05) (Supplementary Fig. S1D).

**Fig. 2:**
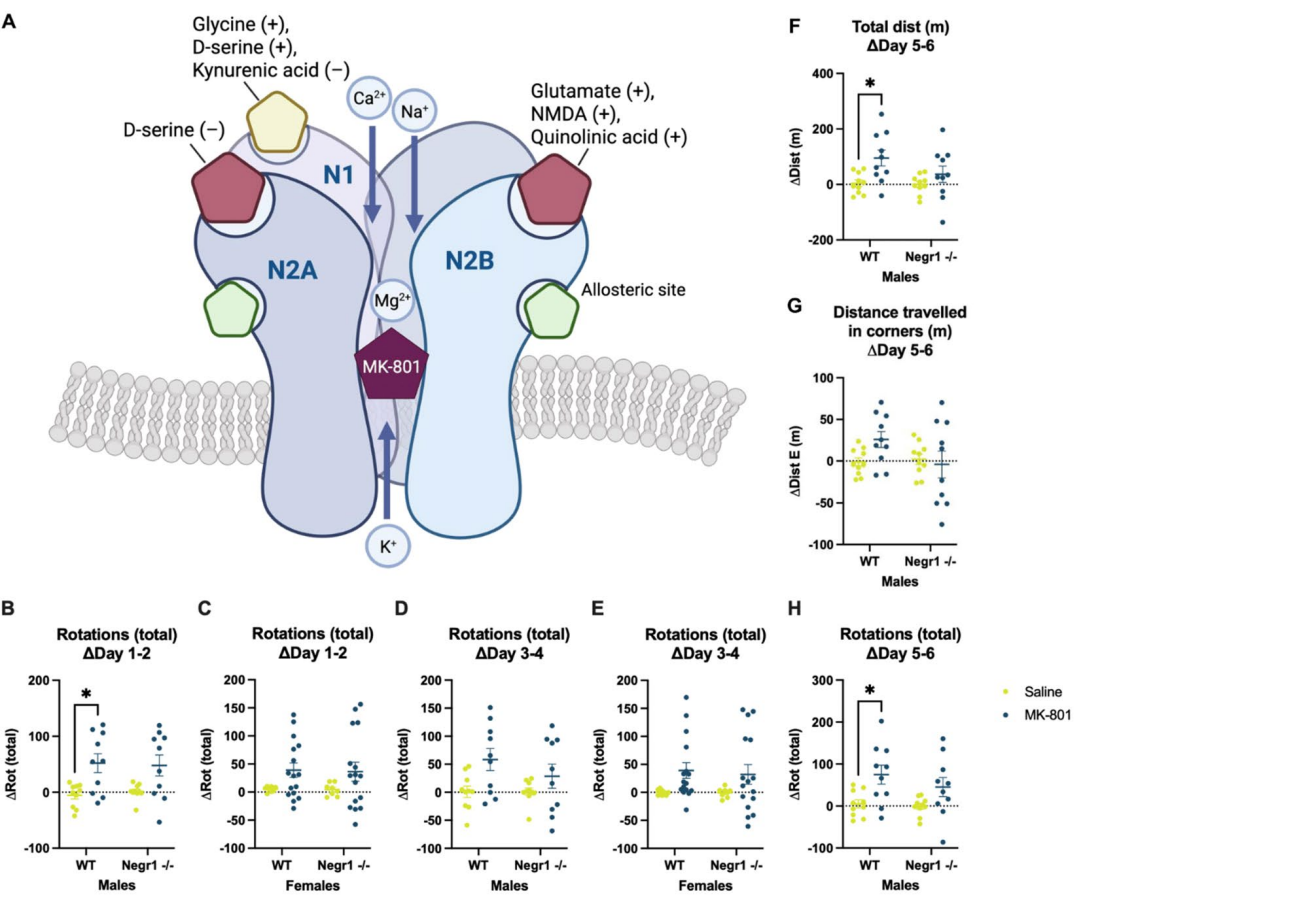
Blunted progression of rapid behavioural tolerance in *Negr1*-deficient mice. **(A)** Schematic representation of the NMDA receptor subunit composition and the ligand-binding sites relevant to this study. The diagram illustrates the receptor as the primary molecular target of MK-801, thereby providing a mechanistic context for the behavioural tolerance data shown in panels B–H. The receptor consists of GluN1 (encoded by *Grin1*), GluN2A (encoded by *Grin2a*), and GluN2B (encoded by *Grin2b*) subunits (other subunits, such as GluN3A, are known but were not investigated here). The GluN1 subunit binds glycine, D-serine, and kynurenic acid, while GluN2A and GluN2B bind glutamate, NMDA, and quinolinic acid. At high concentrations, D-serine may also bind to the GluN2A subunit. MK-801 is a reversible, non-competitive antagonist that blocks the NMDA receptor by binding within its open ion channel. **(B–H)** MK-801-induced stereotypic behaviour and locomotor activity showed a consistent reduction every second day during chronic administration. Delta values for days 1–2 (**B–C**), 3–4 (**D–E**), and 5–6 (**F–H**, available for males only) represent the change in activity observed on each alternate day. *Negr1*^*−/−*^ mice exhibited smaller alterations in behaviour compared to wild-type (WT) controls, indicating a blunted progression of rapid behavioural tolerance. These genotype-dependent fluctuations suggest altered NMDA receptor sensitivity in *Negr1*^*−/−*^ mice. Figure created using BioRender.com.

Interestingly, during repeated MK-801 administration in males, *Negr1*^*−/−*^ mice exhibited a blunted behavioural response over time, suggesting altered sensitivity or tolerance development. Namely, repeated administration of MK-801 elicited distinct locomotor activity patterns in male mice across treatment days, highlighting both genotype-dependent and temporal effects (Figs. 1 and 2, Supplementary Fig. S2-S3).

Across the nine-day observation period, MK-801 treatment produced distinct temporal patterns of locomotor activation in both wild-type (WT) and *Negr1*^*−/−*^ mice. On Day 1, MK-801 elicited a robust stimulatory effect, significantly increasing total distance covered (*p* < 0.0001 for both WT and *Negr1*^*−/−*^ mice), distance covered in the corners (*p* < 0.001 for WT; *p* < 0.05 for *Negr1*^*−/−*^ mice), and the number of rotations (*p* < 0.0001 for both). By Day 2, this response had diminished; total distance remained elevated (*p* < 0.05 for both), but only *Negr1*^*−/−*^ mice continued to display an increased number of rotations (*p* < 0.05), while WT mice showed increased corner activity (*p* < 0.01). On Day 3, a strong stimulatory effect re-emerged, with MK-801 again increasing total distance covered (*p* < 0.00001 for WT; *p* < 0.001 for *Negr1*^*−/−*^), number of rotations (*p* < 0.0001 for WT; *p* < 0.001 for *Negr1*^*−/−*^), and distance covered in corners (*p* < 0.0001 for WT; *p* < 0.05 for *Negr1*^*−/−*^).

By Day 4, the effects had waned, with only small but significant increases observed in total distance (*p* < 0.05 for WT; *p* < 0.01 for *Negr1*^*−/−*^) and corner activity (*p* < 0.01 for both). Rotational activity increased only in *Negr1*^*−/−*^ mice (*p* < 0.01). On Day 5, WT mice exhibited peak activity, with highly significant increases in total distance (*p* < 1 × 10⁻⁷), rotations (*p* < 1 × 10⁻⁶), and corner distance (*p* < 0.0001). In contrast, *Negr1*^*−/−*^ mice showed no such increases, resulting in significant genotype differences across all parameters (distance: *p* < 0.01; corners: *p* < 0.05; rotations: *p* < 0.05). By Day 6, the effects of MK-801 declined markedly in both WT and *Negr1*^*−/−*^ mice, reaching levels comparable to those observed on Days 2 and 4. A gradual attenuation continued through Days 7–9, and by Day 9, activity in both genotypes had dropped significantly from the peak levels seen on Days 3 and 5.

### Effect of repeated treatment with MK-801 (0.2 mg/kg) on locomotor activity in female wild-type and *Negr1*-deficient mice

Female mice displayed a distinct locomotor response profile compared to males, characterised by rapid attenuation of MK-801’s effects (Figs. 1 and 2, Supplementary Fig. S2-S3), but no genotype effect was present.

On Day 1, MK-801 administration significantly increased total distance covered (*p* < 0.001 for both genotypes), the number of rotations (*p* < 0.01), and distance covered in the corners (*p* < 0.001). By Day 2, this response was notably reduced, with significant increases observed only in *Negr1*^*−/−*^ mice (distance: *p* < 0.01; rotations: *p* < 0.05; corners: *p* < 0.05). On Day 3, a partial response was evident, as both genotypes showed increased distance (*p* < 0.05) and corner activity (*p* < 0.01), while only *Negr1*^*−/−*^ mice continued to display an elevated number of rotations (*p* < 0.05). By Days 4 and 5, MK-801’s effects were nearly absent across all parameters, which had declined to saline-control levels, indicating the development of tolerance and leading to the discontinuation of treatment in females.

### Sex differences in response to MK-801

Administering MK-801 resulted in both rapid and general behavioural tolerance, which was measured daily as motor activity in the open field. The drug’s effect diminished every second day in both sexes. General tolerance became evident on day 9 in males and on day 5 in females, leading to sex-specific treatment durations. Notably, the genotype effect was present only in male but not in female mice.

The effects of MK-801 on locomotor activity revealed significant gender-dependent differences, particularly with repeated administrations. These differences became most pronounced by Day 5, prompting a comparative analysis of Days 1 and 5.

In terms of total distance covered, wild-type (WT) males showed a significant increase following MK-801 administration (*p* < 0.05), whereas *Negr1*^*−/−*^ males did not. Among females, MK-801’s effect was significantly reduced by Day 5 in both WT (*p* < 0.001) and *Negr1*^*−/−*^ mice (*p* < 0.0001). Moreover, female mice showed significantly lower locomotor responses compared to their male counterparts (*p* < 0.0001 for WT; *p* < 0.01 for *Negr1*^*−/−*^). A similar pattern emerged for the number of rotations: WT males exhibited increased rotations on Day 5 (*p* < 0.05), while *Negr1*^*−/−*^ males showed no change. In contrast, MK-801 treatment reduced the number of rotations in females, with significant decreases observed in WT (*p* < 0.01) and *Negr1*^*−/−*^ (*p* < 0.001) mice. Female mice also demonstrated significantly fewer rotations than males on Day 5 (*p* < 1 × 10⁻⁵ for WT; *p* < 0.01 for *Negr1*^*−/−*^).

For distance covered in the corners, WT males again showed a significant increase (*p* < 0.05), whereas *Negr1*^*−/−*^ males displayed no detectable change. In females, MK-801 reduced corner distance in *Negr1*^*−/−*^ mice (*p* < 0.0001) and, to a lesser extent, in WT (*p* < 0.05). WT females exhibited a significantly lower response compared to WT males (*p* < 0.01), while no sex difference was detected within the *Negr1*^*−/−*^ group.

### Changes in NMDA-related gene expression due to repeated MK-801 treatment

#### Frontal cortex

**Fig. 3:**
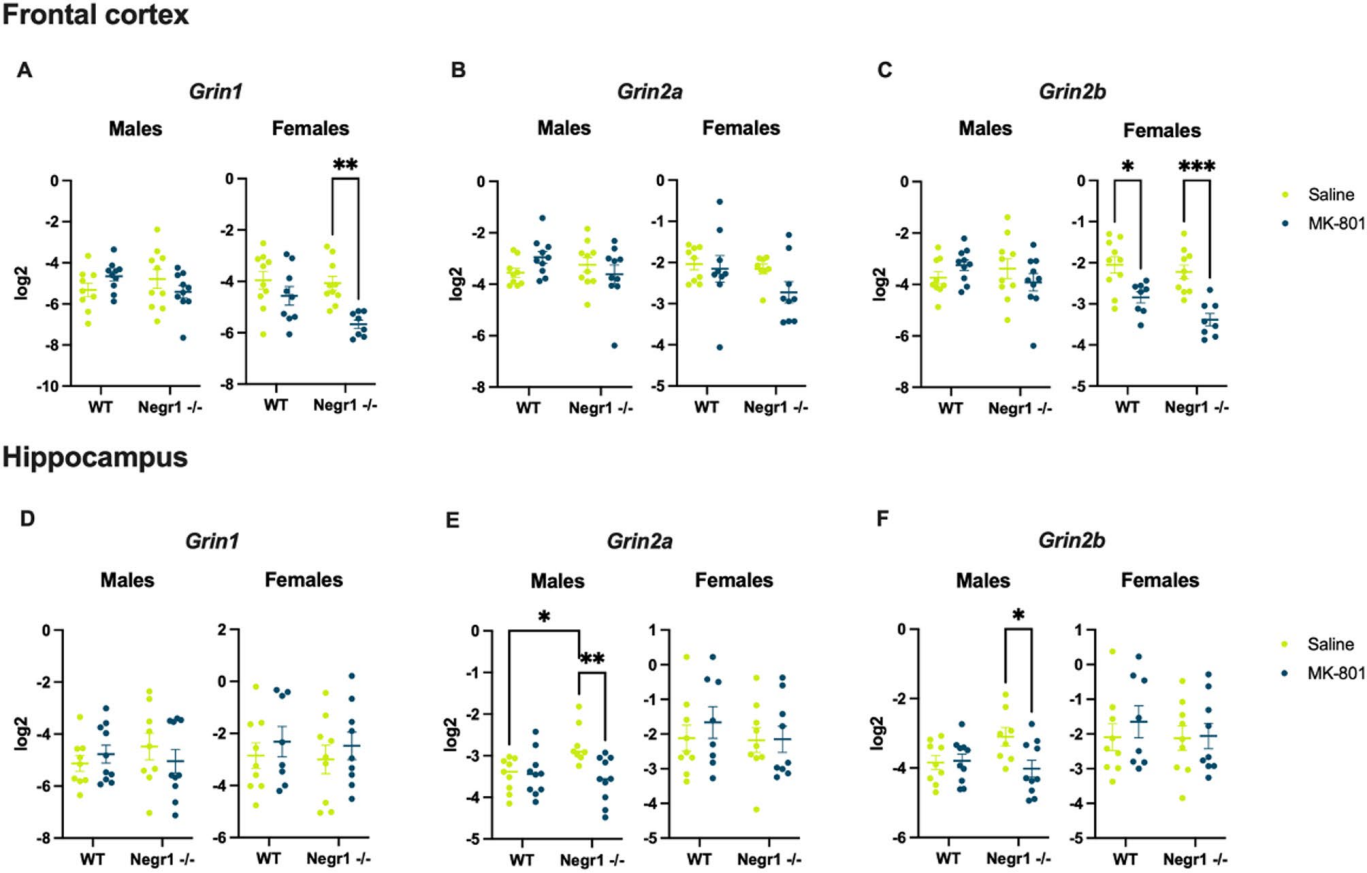
Changes in NMDA-related gene expression in the frontal cortex and hippocampus of mice. The gene expression of glutamate receptor subunit GluN1 encoded by *Grin1* (**A**,** D**), subunit GluN2A encoded by *Grin2a* (**B**,** E**), and GluN2B encoded by *Grin2b* (**C**,** F**) are depicted for both male and female mice. There are four groups in each graph: WT mice injected with physiological solution (saline), WT mice injected with MK-801, *Negr1*-deficient mice injected with physiological solution and *Negr1*-deficient mice injected with MK-801. In the frontal cortex (**A-C**), statistically significant changes were observed only among female mice with *Grin1* and *Grin2b* genes showing sex, genotype and treatment effects. In the hippocampus (D-F), statistically significant changes were observed only among male mice with *Grin2a* and *Grin2b* genes showing sex, genotype, and treatment effects. In each group, *n* = 8–10. Data represent mean ± SEM, ordinary two-way ANOVA (Tukey HSD test). WT – wild-type. * - *p* < 0.05, ** - *p* < 0.01, *** - *p* < 0.001.

In male mice, NMDA-related gene expression tended to be lower than in female littermates. However, MK-801 treatment did not cause significant alterations in gene expression in male mice (Fig. 3A-C).

In the frontal cortex of female mice, the expression of the *Grin2a* gene was unaffected by MK-801 administration (Fig. 3B). For *Grin1*, a significant treatment effect was observed (F₁,₃₃ = 13.12, *p* < 0.001). Post hoc analysis (Tukey HSD test) revealed a significant reduction in *Grin1* expression in *Negr1*-deficient mice (*p* < 0.01), but not in wild-type animals (Fig. 3A).

For *Grin2b*, significant effects of genotype (F₁,₃₁ = 6.06, *p* < 0.05) and treatment (F₁,₃₁ = 31.16, *p* < 0.00001) were identified (Fig. 3C). Post hoc analysis showed a significant reduction in *Grin2b* expression in wild-type (*p* < 0.01) and *Negr1*-deficient mice (*p* < 0.001).

For *Srr*, MK-801 treatment had a significant effect (F₁,₃₃ = 6.89, *p* < 0.05), but post hoc analysis did not reveal specific group differences (Supplementary Fig. S5).

#### Hippocampus

In female mice, MK-801 treatment did not result in significant changes in NMDA-related gene expression in the hippocampus (Fig. 3D-F).

In male mice, a significant change was observed for *Grin2a* expression, with a treatment effect (F₁,₃₃ = 6.08, *p* < 0.05) and genotype × treatment interaction (F₁,₃₃ = 7.12, *p* < 0.01). Post hoc analysis showed a significant increase in *Grin2a* expression in *Negr1*-deficient mice who were given physiological solution compared to the mice who received MK-801 (*p* < 0.001) and wild-type mice (*p* < 0.05) (Fig. 3E). Regarding *Grin2b* expression, a significant change was seen with a genotype × treatment interaction (F₁,₃₃ = 4.56, *p* < 0.05). The levels of *Grin2b* expression were increased in *Negr1*-deficient mice (*p* < 0.05) who were given physiological solution compared to the mice who received MK-801 (Fig. 3F).

#### Ventral striatum

We also measured the expression of these genes in the ventral striatum (Supplementary Fig. S4) and measured the *Srr* expression (Supplementary Fig. S5), but found no significant differences between groups.

#### Correlational analysis of kynurenine pathway metabolites, Tryptophan and glutamate across brain regions and blood plasma

In the correlation analysis, we compared WT and *Negr1*-deficient mice, both male and female. Analysis included seven metabolites, which we determined most relevant to this paper’s topic. Tryptophan and kynurenine were chosen because they are the direct precursors to kynurenic acid and quinolinic acid, which are the NMDA receptor co-antagonist and co-agonist, respectively. Picolinic acid and xanthurenic acid were chosen due to their antioxidant properties and previously found connections to mental disorders^40,41^. In addition, we looked at glutamate to better understand the interaction between the kynurenine pathway and glutamate signalling. The data was gathered from blood plasma and four brain regions: frontal cortex, hippocampus, hypothalamus and ventral striatum (Supplementary Fig. S6 and Fig. S7, Supplementary tables S2-S5).

#### Similarities between groups

Analysis revealed several conserved and biologically meaningful correlations across sexes and genotypes, indicating stable metabolic interactions within the kynurenine–glutamate network (Supplementary Tables S2–S5). For example, xanthurenic acid in the hippocampus was positively correlated with quinolinic acid in the same region in both female *Negr1*-deficient (*r* = 0.76, *p* < 0.01) and female wild-type (*r* = 0.56, *p* < 0.05) groups, demonstrating a consistent association across genotypes. More broadly, quinolinic acid and xanthurenic acid displayed positive correlations across multiple regions, including the frontal cortex (*r* = 0.84, *p* < 0.001) and ventral striatum (*r* = 0.78, *p* < 0.01), underscoring a preserved coupling between these metabolites.

Similarly, glutamate and quinolinic acid in the frontal cortex were positively correlated in all groups (Supplementary Tables S2–S5), supporting a core link between excitatory neurotransmission and kynurenine pathway activity. Additional associations between xanthurenic acid and glutamate were also observed, particularly in *Negr1*-deficient mice (e.g., frontal cortex males *r* = 0.57, *p* < 0.05; females *r* = 0.91, *p* < 1.36 × 10⁻⁵), suggesting a mutation-specific link between glutamate and xanthurenic acid metabolism. Xanthurenic acid in blood plasma additionally correlated with several kynurenine metabolites in males. In male wild-type mice, plasma xanthurenic acid correlated positively with kynurenine in the ventral striatum (*r* = 0.55, *p* < 0.05) and negatively with kynurenic acid in the ventral striatum (*r* = − 0.54, *p* < 0.05). In male *Negr1*-deficient mice, plasma xanthurenic acid correlated inversely with kynurenine in plasma (*r* = − 0.56, *p* < 0.05) and positively with glutamate in the frontal cortex (*r* = 0.57, *p* < 0.05) — indicating that while the direction of these relationships varied, the involvement of xanthurenic acid remained a recurring feature across groups.

#### Differences between groups

Distinct patterns emerged when comparing male and female groups. Male mice exhibited a higher number of significant correlations involving xanthurenic acid in plasma, while female mice showed a greater emphasis on xanthurenic acid correlations in the hippocampus (Supplementary tables S2-S5). Notably, female *Negr1*-deficient mice demonstrated particularly strong xanthurenic acid-related associations, including a robust correlation between quinolinic acid and xanthurenic acid in the frontal cortex (*r* = 0.86, *p* < 0.001) and a similarly strong association between glutamate and xanthurenic acid (*r* = 0.81, *p* = 7.5 × 10⁻⁴).

When comparing *Negr1*-deficient and wild-type mice groups, several differentiating features became apparent (Supplementary tables S2-S5). Male wild-type mice displayed characteristic tryptophan–kynurenine pathway relationships involving plasma xanthurenic acid and ventral striatum metabolites, such as positive correlations between xanthurenic acid in plasma and kynurenine in the ventral striatum (*r* = 0.55, *p* = 0.042) and a negative correlation with kynurenic acid in the ventral striatum (*r* = − 0.54, *p* = 0.045). In contrast, male *Negr1*-deficient mice gained additional cross-compartment associations linking plasma xanthurenic acid to both kynurenine and glutamate, with xanthurenic acid and kynurenine in plasma showing a negative correlation (*r* = − 0.56, *p* = 0.046) and xanthurenic acid in plasma and glutamate in frontal cortex a positive one (*r* = 0.57, *p* = 0.041). In addition, kynurenic acid showed the fewest significant correlations in female *Negr1*-deficient mice, suggesting a selective reduction in KYNA-related interactions in this group.

#### Changes in the kynurenine pathway and glutamate levels of *Negr1*-deficient mice

**Fig. 4:**
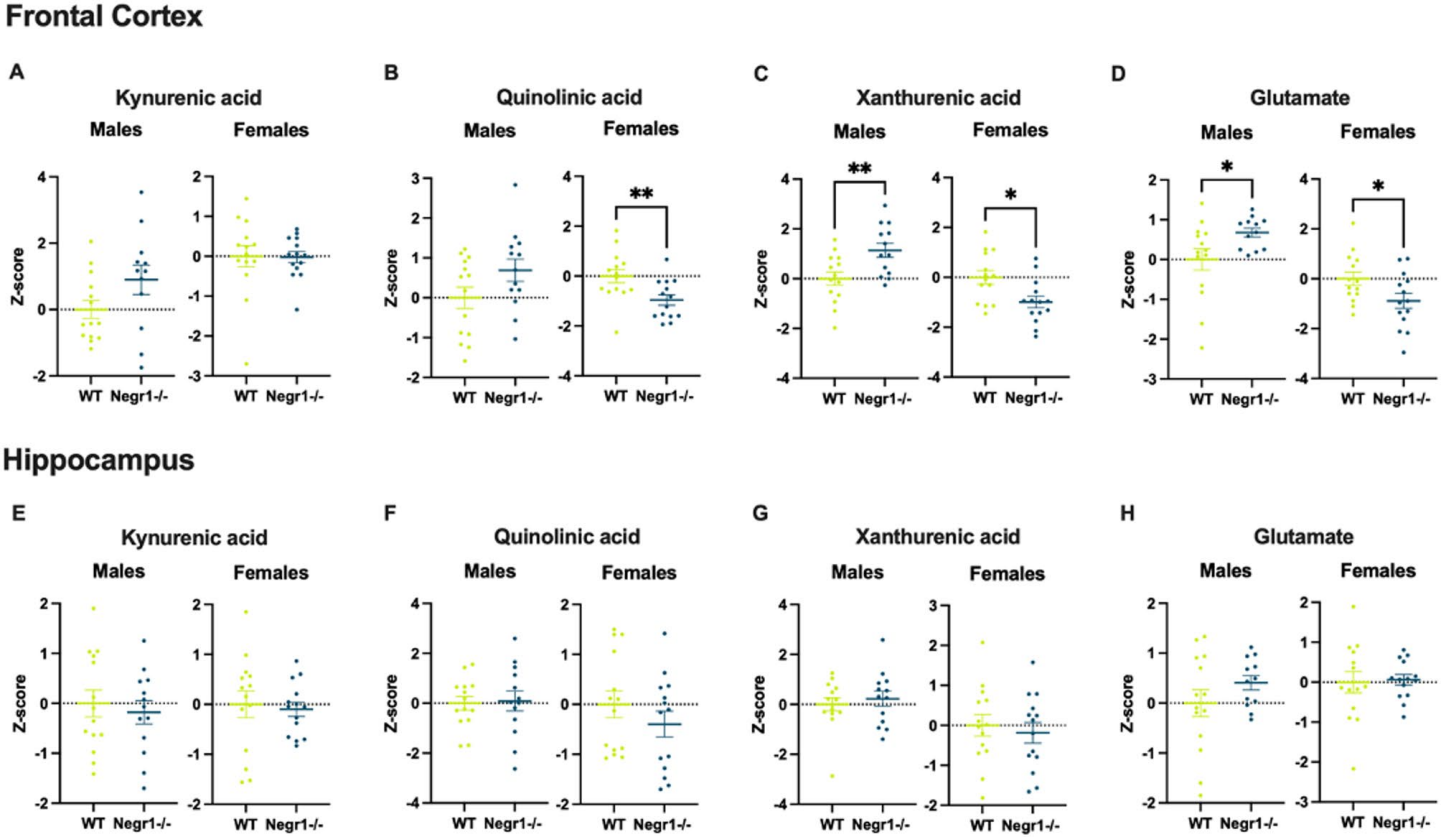
Changes in the frontal cortex and hippocampus metabolite levels of *Negr1*-deficient mice (Cohort 2). The figure depicts z-scores of the kynurenic acid (**A**,** E**), quinolinic acid (**B**,** F**), xanthurenic acid (**C**,** G**) and glutamate (**D**,** H**) levels in wild-type and *Negr1*-deficient male and female mice. Results show significant differences in the xanthurenic acid and glutamate levels between *Negr1*^*−/−*^ and WT male and female mice in the frontal cortex, with the levels being elevated in *Negr1-/-* males and diminished in *Negr1*^*−/−*^ females. There was also a statistically significant decline in the quinolinic acid levels of *Negr1*^*−/−*^ female mice. No statistically significant differences were observed in the measured metabolite levels between WT and *Negr1*^*−/−*^ mice in the hippocampus. Data represent mean ± SEM, unpaired t-test results, *n* = 12–14. WT – wild type. * - *p* < 0.05, ** - *p* < 0.01.

**Fig. 5:**
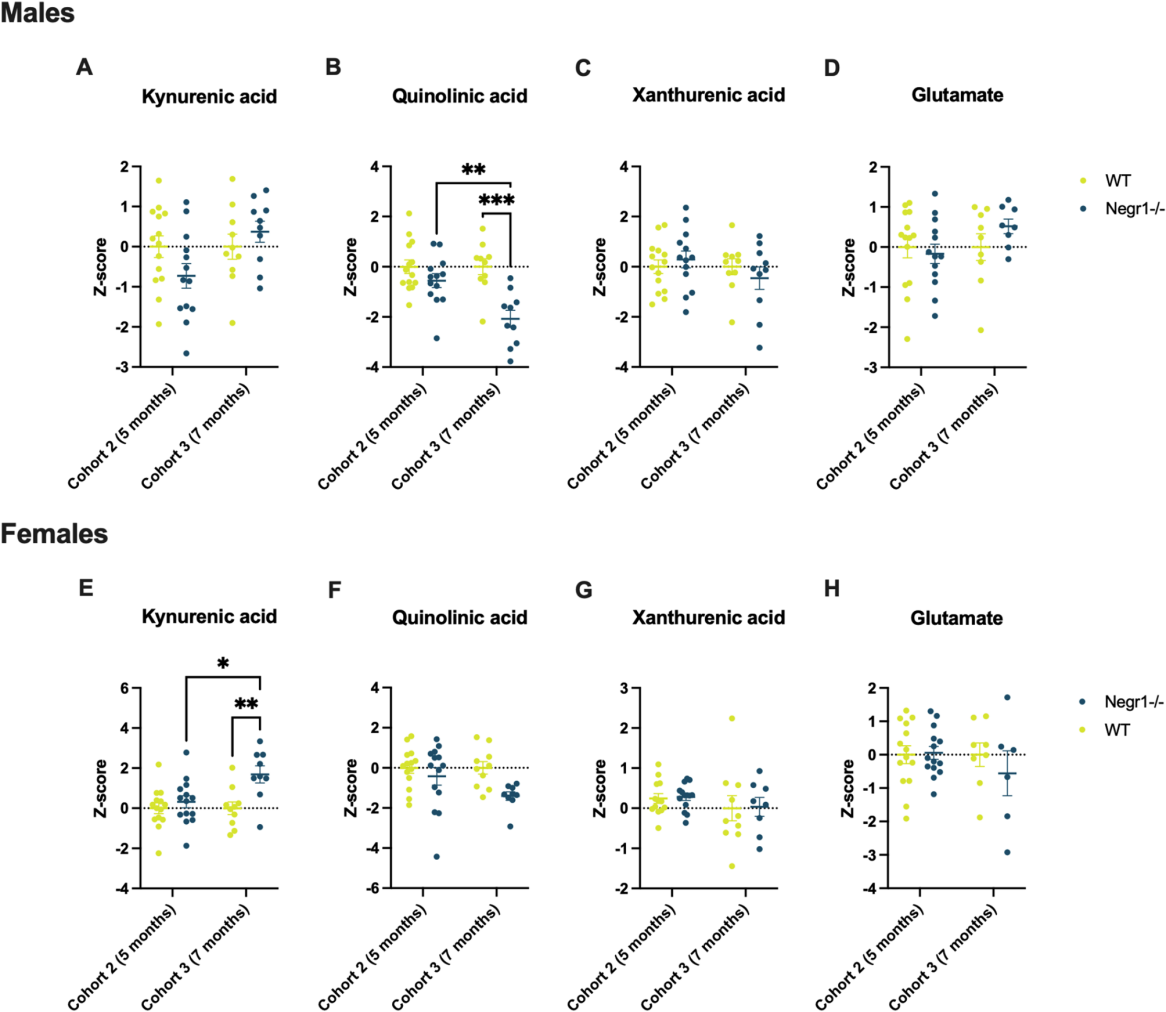
Changes in the blood plasma metabolite levels of *Negr1*-deficient mice (Cohorts 2 and 3). The figure depicts z-scores of the kynurenic acid (**A**,** E**), quinolinic acid (**B**,** F**), xanthurenic acid (**C**,** G**) and glutamate (**D**,** H**) levels in wild-type and *Negr1*-deficient male and female mice. Shown are data from cohort 2 (5-month-olds) and cohort 3 (7-month-olds). There was a significant decrease in the quinolinic acid level among the older *Negr1*^*−/−*^ male mice and an increase in the kynurenic acid level of older *Negr1*^*−/−*^ female mice. Xanthurenic acid and glutamate levels remained approximately the same for both genders. Data represent mean ± SEM, 2-way ANOVA results (Tukey HSD test), n = (6)8–14. WT – wild type. * - *p* < 0.05, ** - *p* < 0.01, *** - *p* < 0.001.

As a result of the correlation analysis, we focused on kynurenic acid, quinolinic acid, xanthurenic acid and glutamate in the frontal cortex, hippocampus and blood plasma (Figs. 4 and 5). We also looked at the levels of these metabolites in hypothalamus and ventral striatum, but did not see as many significant differences (Supplementary Fig. S8 - Fig. S17).

There was no statistically significant difference in the kynurenic acid and quinolinic acid levels between WT and *Negr1*-deficient male mice in the frontal cortex, although there seemed to be a trend for an increase among mutant mice compared to the WT controls (Fig. 4A and B). The xanthurenic acid (*p* < 0.01) and glutamate levels (*p* < 0.05), however, were significantly increased in the *Negr1*-deficient male mice (Fig. 4C and D).

The level of kynurenic acid remained unchanged for the female mice in the frontal cortex (Fig. 4A), but contrary to the male mice, the levels of quinolinic acid (*p* < 0.01), xanthurenic acid (*p* < 0.01) and glutamate (*p* < 0.05) were considerably reduced (Fig. 4B-D).

The levels of measured metabolites in the hippocampus of *Negr1*-deficient male and female mice were not significantly altered compared to the wild-type mice (Fig. 4E-H).

The blood plasma analysis included two cohorts to estimate the dynamics of the biochemical shifts during ageing: cohort 2 consisted of 5-month-old mice, and cohort 3 of 7-month-old mice (Fig. 5). Our results indicate that older mice were more strongly influenced by genotype. Specifically, male *Negr1*^*−/−*^ mice showed a significant decrease in quinolinic acid levels (*p* < 0.001) (Fig. 5B), and female *Negr1*^*−/−*^ mice exhibited a significant increase in kynurenic acid levels (*p* < 0.05) (Fig. 5E) compared to age-matched wild-type controls (5-month-olds). The reduction in quinolinic acid levels in male mice remained significant when the two age groups were combined (*p* < 0.01), whereas the increase in kynurenic acid in females did not (Supplementary Fig. S14). Xanthurenic acid and glutamate levels remained relatively unchanged across sex, age, and genotype groups.

## Discussion

This study is the first to demonstrate a link between *Negr1*, NMDA receptor function, and kynurenine pathway metabolites, resulting in significant behavioural alterations. While *NEGR1* has been associated with various psychiatric disorders^42^, we extended this research using MK-801, a non-competitive NMDA receptor antagonist, to model glutamatergic imbalances observed in neuropsychiatric and neurodegenerative conditions^24–26^.

Behavioural analyses revealed significant differences between saline- and MK-801-treated mice. Acute MK-801 administration elicited a heightened motor response in drug-naive *Negr1*-deficient males compared to wild-type controls. However, with repeated exposure, *Negr1*-deficient males displayed a blunted response, indicating altered NMDA receptor sensitivity or tolerance development. The most pronounced changes were observed in total distance covered, distance covered in corners, and rotational behaviour. MK-801–induced hyperlocomotion is attributed to its action on GABAergic interneurons; NMDA receptor blockade reduces inhibitory tone and indirectly enhances excitatory output^43^. The exaggerated initial response in *Negr1*-deficient mice may reflect a baseline reduction in GABAergic tone^5,44^, amplifying the disinhibitory effects of MK-801, consistent with prior findings of disrupted excitatory and inhibitory balance in models of psychiatric disease^45^. Recent evidence suggests a mechanistic explanation for this phenotype: NEGR1 promotes clearance of the GABA-synthesising enzyme GAD65 from the plasma membrane, thereby maintaining inhibitory synapse density and GABAergic tone^13^. Loss of NEGR1 reduces the number of GABAergic synapses and synaptic GABA levels, shifting the excitation–inhibition balance toward excessive excitatory drive. This mechanism could sensitise neuronal networks to NMDA receptor blockade, explaining the heightened acute response to MK-801 observed in *Negr1*-deficient mice.

An unexpected zig-zag pattern in behavioural responsiveness emerged, marked by reduced activity every other day, suggesting the rapid development of behavioural tolerance to daily MK-801 administration. The underlying mechanism remains unclear but may involve residual drug accumulation due to MK-801’s long half-life^26^ or transient NMDA receptor desensitisation^46^. Repeated exposure could trigger rapid yet reversible neuroadaptive processes, such as receptor upregulation or alterations in downstream signalling^16^. After a brief recovery period, receptor sensitivity may reset, restoring responsiveness. Although this pattern was evident in both genotypes, *Negr1*-deficient mice showed a stronger progression of tolerance, indicating altered NMDA receptor sensitivity.

In addition, *Negr1*-deficient male mice exhibited a stronger acute response to MK-801 but developed tolerance more rapidly with repeated dosing. Behavioural suppression — seen as reduced locomotion and stereotypy — diminished more quickly in *Negr1*-deficient mice compared to wild-type controls, particularly across treatment intervals (delta days 1–2, 3–4, and 5–6; Fig. 2 and supplementary Fig. S3).

These findings suggest that *Negr1* deficiency alters NMDA receptor function or regulation, potentially due to increased receptor availability^4^. Elevated baseline NMDA receptor density may heighten initial MK-801 sensitivity while accelerating desensitisation or downstream adaptations during repeated exposure. Overall, the data indicate dysregulated NMDA receptor dynamics in *Negr1*-deficient mice, influencing both acute responsiveness and the trajectory of tolerance development.

At the molecular level, our data indicate a complex, sex- and region-specific modulation of NMDA receptor subunit expression. Previous studies have shown that receptors with a higher GluN2B-to-GluN2A (*Grin2b*-to-*Grin2a*) ratio are more susceptible to quinolinic acid–induced neurotoxicity due to their predominant expression in immature neurons and extrasynaptic sites, where they can promote excitotoxicity^47–49^. In the present study, a similar pattern appeared in the frontal cortex of adult female mice but was not observed in the hippocampus or in male mice. However, some studies have reported contrasting findings — highlighting a critical role for GluN2B in intracellular signalling and excitotoxicity and suggesting that both GluN2A and GluN2B subunits contribute equally to extrasynaptic signalling^50,51^. Furthermore, we found that female *Negr1*-deficient mice treated with MK-801 exhibited reduced expression of GluN1 (*Grin1*) in the frontal cortex. Together, these findings suggest a sex- and brain region-specific interaction between *Negr1* deficiency and NMDA receptor regulation.

In male mice, expression levels of NMDA receptor subunit genes in the frontal cortex did not differ significantly between genotypes. In contrast, in the hippocampus, *Grin2a* and *Grin2b* were significantly upregulated in *Negr1*-deficient mice treated with physiological solution compared to wild-type controls. This finding aligns with earlier evidence of increased NMDA receptor binding density in the hippocampus of *Negr1*-deficient animals^4^, suggesting elevated baseline receptor availability in this brain region under non-challenged conditions. Interestingly, MK-801 administration normalised the expression of these subunits to levels comparable with wild-type controls. This pattern may reflect a compensatory mechanism, wherein *Negr1*-deficient mice upregulate NMDA receptor subunits to counterbalance impaired receptor function or altered inhibitory signalling. Alternatively, increased expression could serve to maintain excitatory-inhibitory homeostasis in the context of disrupted GABAergic tone. In addition to our current findings concerning excitatory NMDA receptors, NEGR1 has been implicated in AMPA receptor trafficking and dendritic spine maturation^14^, suggesting that its loss may impair excitatory synaptic organisation and plasticity. Such disruption could lead to compensatory upregulation of NMDA receptor subunits as the system attempts to stabilise synaptic strength. MK-801 treatment may override this compensatory adaptation by saturating receptor activity and externally shifting the excitatory-inhibitory balance. However, previous studies have shown that overexpression of *Grin2a* and *Grin2b* can exacerbate neuronal vulnerability^51^, and GluN2A overexpression has been associated with impaired synaptic structure and function^52^. In contrast, GluN2B overexpression has been linked to improved learning and memory^53–55^. These contrasting outcomes highlight the complexity of NMDA receptor regulation and emphasise the need for further research to determine whether such subunit overexpression is neuroprotective or detrimental in the context of *Negr1* deficiency. Although gene expression was assessed after behavioural adaptation to MK-801, this reflects a typical compromise in longitudinal study designs. Future studies could build on these findings by targeting more specific time points — such as day 5 in males and day 3 in females — when behavioural phenotypes diverge most clearly. These adjustments would help to refine the temporal resolution of gene expression dynamics and strength causal interpretations.

One of the most notable findings of this study was the emergence of clear sex differences, underscoring the importance of including both male and female animals in neurobiological research^56,57^. Previous studies have reported sex-specific differences in NMDA receptor function and responses to NMDA receptor antagonists^58–60^. Our results extend these observations by showing that sex differences in *Negr1*-deficient mice are evident not only in behaviour but also in kynurenine pathway metabolites and glutamate levels. Over the course of five days, wild-type males displayed a progressive increase in locomotor activity following repeated MK-801 administration, indicative of sensitisation. In contrast, *Negr1*-deficient males showed minimal behavioural change, suggesting altered receptor responsiveness or adaptation. Female mice, regardless of genotype, exhibited more rapid tolerance and sensitisation to MK-801, reflected by a decline in locomotor activity over time. These findings highlight a dynamic interplay between sex, genotype, and NMDA receptor function and point to sex-specific mechanisms of behavioural plasticity in response to glutamatergic disruption.

Although we anticipated that kynurenic acid (KYNA) and quinolinic acid (QUIN) levels would directly influence NMDA receptor function in *Negr1*-deficient mice, our findings suggest a more nuanced relationship. While levels of kynurenine pathway metabolites were altered in the *Negr1*-deficient group, these changes did not appear to drive NMDA receptor-related behavioural outcomes directly. This may indicate that NMDA receptor function was maintained through compensatory mechanisms involving other co-agonists or modulatory systems. In addition, correlation analyses revealed that kynurenine pathway metabolite profiles were region-specific. The frontal cortex was the most affected by *Negr1* deficiency, whereas other brain regions exhibited few significant changes (Supplementary Fig. S6–S7; Supplementary Table S2–S5; Supplementary Fig. S8–S17). These findings emphasise the importance of spatial context when studying neuroimmune-metabolic interactions and suggest that the impact of *Negr1* on kynurenine metabolism may be anatomically selective. Furthermore, the effects of *Negr1* deficiency became more pronounced with age, with older mice showing stronger genotype-related shifts in kynurenine pathway metabolites. This suggests that ageing may exacerbate or unmask metabolic consequences of *Negr1* deficiency.

### Limitations

Despite our best efforts, this study has some limitations that should be addressed. Gene expression was assessed after behavioural adaptation to MK-801, limiting the understanding of gene expression during the behavioural experiments. In the future, gene expression should be assessed on the 3rd day for female and 5th day for male mice as these were the days with the biggest statistical significance between the studied groups. Furthermore, the present study examined transcript-level changes regarding NMDA receptors without direct assessment of protein abundance or receptor function, which will require complementary biochemical or electrophysiological approaches. Finally, due to methodological limitations, we could not study NMDA receptor sensitivity and kynurenine pathway metabolites in the same mice cohort, which limits the ability to establish direct integration of molecular and behavioural outcomes.

### Implications for future research

Altogether, these findings identify *Negr1* as a key modulator of glutamatergic signalling, with potential implications for understanding individual susceptibility to conditions involving NMDA receptor dysfunction. The observed sex- and region-specific effects indicate that *Negr1*-related pathways may influence excitatory–inhibitory balance through distinct regulatory mechanisms across neural circuits. These results provide a framework for exploring how *Negr1*-dependent modulation of NMDA receptor function interacts with metabolic processes, particularly the kynurenine pathway, to influence neuronal and behavioural outcomes. Mechanistically, NEGR1 appears to function as a synaptic organiser that coordinates inhibitory and excitatory signalling through regulation of GAD65 turnover and AMPA receptor trafficking^13,14^. Its absence, therefore, likely disturbs the molecular scaffolding required for balanced neurotransmission, leading to maladaptive plasticity and altered NMDA receptor dynamics observed in this study. By establishing a link between *Negr1*, NMDA receptor dynamics, and kynurenine metabolism, our data position *Negr1* as a useful entry point for probing metabolic–synaptic interactions in neuropsychiatric disease models. These results therefore offer a good foundation and testable hypotheses for research into *Negr1*-related neurobiology and its contribution to glutamate-driven behavioural phenotypes. A deeper understanding of these mechanisms may help identify novel therapeutic targets for disorders characterised by glutamatergic dysregulation.

## Conclusion

This study demonstrates that *Negr1* deficiency leads to pronounced, sex-specific alterations in glutamatergic signalling, behavioural responses to NMDA receptor antagonism, and kynurenine pathway metabolism. These effects were both brain region– and sex–dependent, underscoring the importance of considering biological sex and genetic background when modelling neuropsychiatric disorders. Our findings suggest that *Negr1* influences NMDA receptor availability and dynamics, contributing to altered sensitivity and tolerance to glutamatergic disruption. Moreover, the observed region-specific changes in kynurenine metabolites highlight a possible link between neuroimmune metabolism and glutamatergic function in the *Negr1*-deficient brain. Taken together, these results provide novel insights into the neurobiological mechanisms associated with *Negr1* and support its relevance as a molecular node connecting genetic risk, glutamate dysregulation, and sex-dependent vulnerability in psychiatric disorders. Targeting *Negr1*-related pathways may open new avenues for understanding and eventually mitigating glutamate-related dysfunction in mental illness.

## Methods

### Animals

Adult male and female wild-type (WT) mice and their homozygous *Negr1*-deficient littermates (*Negr1*^−/−^), previously generated and described by Lee et al. (2012), were used in this study^61^. All mice were on an F2 hybrid background: ((129S5/SvEvBrd × C57BL/6 N) × (129S5/SvEvBrd × C57BL/6 N)). The mouseline was maintained on a mixed background and no further backcrossing was performed to avoid the *congenic footprint*—retention of embryonic stem cell–derived chromosomal segments flanking the targeted allele—which can confound phenotype interpretation^62^. Animals were group-housed (10 per cage) in standard laboratory cages (42.5 × 26.6 × 15.5 cm) under controlled environmental conditions (22 ± 1 °C; 12:12 h light/dark cycle, with lights off at 19:00). Each cage contained a 2 cm layer of aspen bedding and 0.5 L of aspen nesting material (Tapvei, Paekna, Estonia), which were changed weekly. Food pellets (R70, Lactamin AB, Kimstad, Sweden) and water were provided *ad libitum*. Breeding and maintenance were carried out at the animal facility of the Institute of Biomedicine and Translational Medicine, University of Tartu, Estonia.

**Fig. 6:**
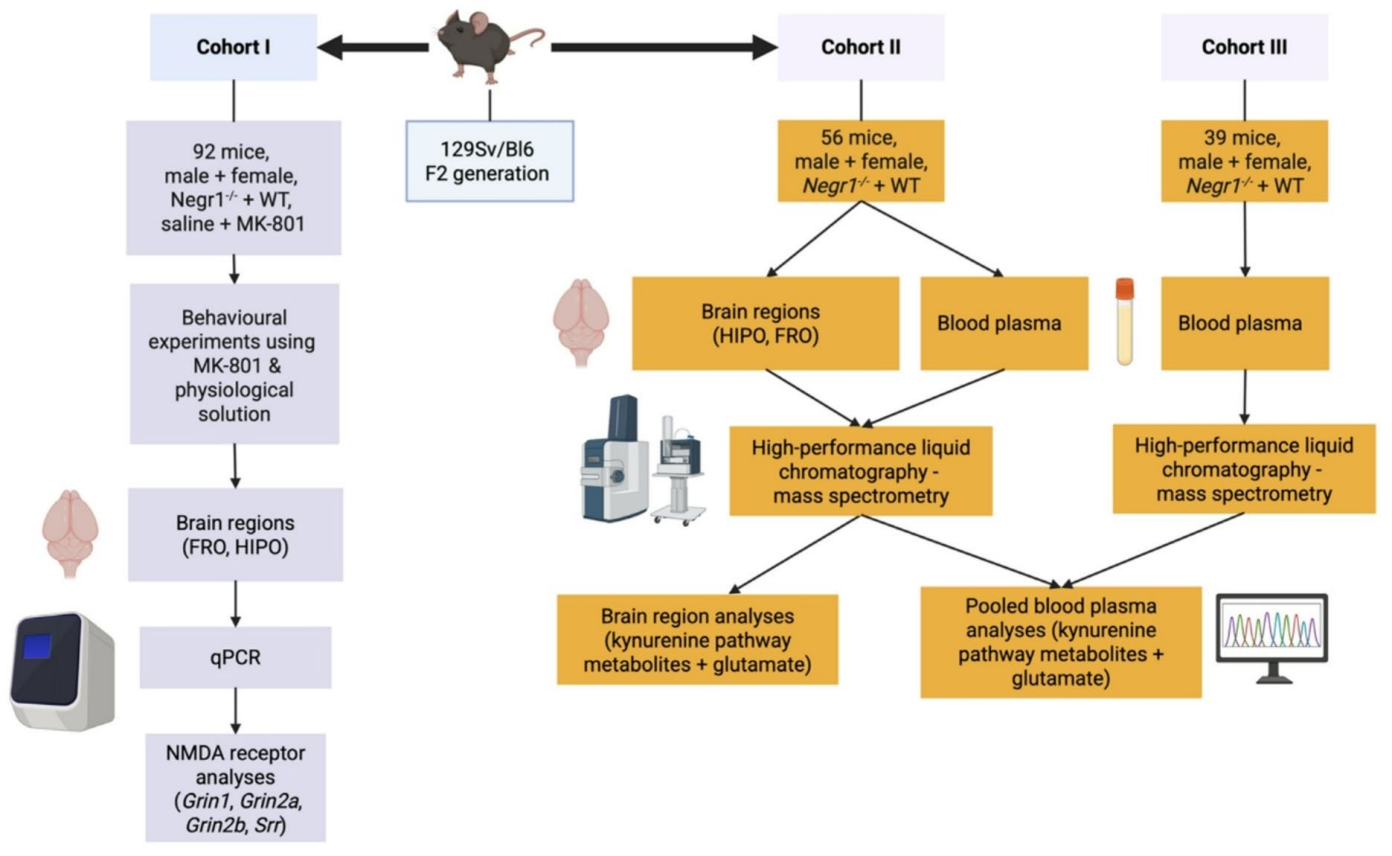
General description of the study. Created with BioRender.

All behavioural testing was conducted between 8:00 a.m. and 5:00 p.m. Prior to testing, mice were kept in group housing conditions to minimise stress.

Three separate mouse cohorts were used in this study (Fig. 6):

#### Cohort 1

Included 2-month-old male and female mice, with equal representation of WT and *Negr1*^*−/−*^ genotypes. The age of the mice was chosen to match the age of mice used in Singh et al. (2018) where the differential receptor sensitivity to MK-801 was shown in vitro in *Negr1*^*−/−*^ hippocampal slices. Half of the mice in each genotype group received the NMDA receptor antagonist MK-801, while the remaining animals received physiological solution (saline). Body weight was monitored throughout the experiment and before each injection. No significant changes in body weight were observed during the experimental period; therefore, body weights measured at the end of the experiment, immediately before brain dissection and blood collection, are reported. The mean body weight of female WT (saline) mice was 23.8 (SD ± 1.5) g, 22.7 (± 2.1) g for female WT (MK-801) mice, 22.7 (± 1.4) g for female *Negr1*^*−/−*^ (saline) mice, 21.3 (± 2.5) g for female *Negr1*^*−/−*^ (MK-801) mice. The mean body weight of male WT (saline) mice was 27.1 (SD ± 3.0) g, 26.4 (± 2.5) g for male WT (MK-801) mice, 27.4 (± 2.6) g for male *Negr1*^*−/−*^ (saline) mice, 26.8 (± 2.9) g for male *Negr1*^*−/−*^ (MK-801) mice. This cohort was used to investigate the role of NMDA receptor function in a schizophrenia spectrum disorder model.

#### Cohort 2

Comprised 5-month-old male and female WT and *Negr1*^*−/−*^ mice. The mean body weight of female WT mice was 23.7 (SD ± 2.3) g, 21.8 (± 1.6) g for female *Negr1*^*−/−*^ mice, 32.2 (± 3.4) g for male WT mice and 29.8 (± 2.4) g for male *Negr1*^*−/−*^ mice. Brain tissues and blood plasma were collected for analysis of tryptophan pathway metabolites and glutamate.

#### Cohort 3

Included 7-month-old male and female mice of both genotypes. The mean body weight of female WT mice was 24.8 (SD ± 1.9) g, 24.9 (± 3.7) g for female *Negr1*^*−/−*^ mice, 32.3 (± 2.3) g for male WT mice and 30.8 (± 2.6) g for male *Negr1*^*−/−*^ mice. These mice were handled identically to those in Cohort 2, although at a different time point. Blood plasma was collected for additional tryptophan pathway metabolites and glutamate analysis. Older mice were used to estimate the dynamics of the biochemical shifts during ageing.

All animal procedures were carried out by licensed professionals in accordance with the European Communities Directive (2010/63/EU) and approved by the Laboratory Animal Centre at the Institute of Biomedicine and Translational Medicine, University of Tartu, Estonia. The study was conducted under a permit from the Estonian National Board of Animal Experiments (Permit No. 150, 27 September 2019). We confirm this study is reported in accordance with the ARRIVE (Animal Research: Reporting of In Vivo Experiments) guidelines as outlined at https://arriveguidelines.org (Supplementary ARRIVE guidelines checklist.)

### MK-801 treatment

In the dose response experiment, mice received MK-801 (dizocilpine) in three different dosages: 0.1 mg/kg, 0.2 mg/kg and 0.4 mg/kg (Supplementary Fig. S1). A concentration of 0.2 mg/kg was chosen for the chronic MK-801 experiment. All participating mice received an intraperitoneal injection. Control mice received a corresponding injection of physiological solution (saline).

### Open field test

Locomotor activity of individual mice was measured with the illumination level of 450 lx for 30 min in soundproof photoelectric motility boxes (44.8 × 44.8 × 45 cm) connected to a computer (TSE, Technical & Scientific Equipment GmbH, Berlin, Germany). The floor of the testing apparatus was cleaned with 70% ethanol and dried thoroughly after each mouse. The system automatically registered the movement of the animal and the time it took to do all the following activities: the distance covered in total, and in corners of the box, the number of rearings, rotations (clockwise + counterclockwise) and corner visits.

During the behavioural experiment period, all animals were monitored daily for signs of weight loss and injuries that could potentially be caused by group housing. After the behavioural experiments, mice were euthanised by rapid decapitation using surgical scissors as the primary method, allowing the collection of both trunk blood and brain tissues. No anaesthesia was used as it could confound the interpretation of downstream molecular analyses, including qPCR and mass spectrometry.

### RT-qPCR analysis in mouse brain areas

Gene expression was determined by two-step RT-qPCR in the frontal cortex, hippocampus and ventral striatum. These regions were selected because they are implicated in psychiatric disorders, exhibit high levels of NMDA receptor expression, and have previously been shown to display alterations in excitatory and/or inhibitory neurotransmission in *Negr1*-deficient mice. Total RNA was extracted from each tissue sample by using Trizol reagent (Invitrogen) according to the manufacturer’s protocol. First-strand cDNA was synthesised by using FIREScript^®^ RT cDNA synthesis MIX with Oligo (dT) and Random primers (Solis BioDyne, Tartu, Estonia) according to the manufacturer’s protocol.

In qPCR, four NMDA receptor subunit-related genes were studied: glutamate ionotropic receptor NMDA type subunit 1 (GluN1, gene *Grin1*), glutamate ionotropic receptor NMDA type subunit 2a (GluN2A, gene *Grin2a*), glutamate ionotropic receptor NMDA type subunit 2b (GluN2B, gene *Grin2b*) and serine racemase (Srr, gene *Srr*). *HPRT* (hypoxanthine guanine phosphoribosyltransferase) was used as a housekeeper gene. The same primers have been previously described in Varul et al., 2021^63^. Primer sequences can be found in Supplementary Table S6. For qPCR, all reactions were performed in a final volume of 10 µL, using 5 ng of cDNA and HOT FIREPol^®^ EvaGreen^®^ qPCR Supermix (Solis BioDyne). Every reaction was made in four parallel replicates to minimise possible errors. ABI Prism 7900HT Sequence Detection System with ABI Prism 7900 SDS 2.4.2 software (Applied Biosystems) was used for qPCR detection. Data in the Figures is presented on a linear scale, calculated as 2^−ΔCT^, where ΔCT is the difference in cycle threshold (CT) between the target genes and the housekeeper gene.

### Measurement of biomarkers

From all the second and third cohorts’ mice’s blood plasma, the levels of 8 different tryptophan pathway metabolites and glutamate were measured using high-performance liquid chromatography-mass spectrometry (Waters Xevo TQ-XS with Acquity H-class UPLC). From the second cohort, the same metabolite levels were also measured in the frontal cortex, hippocampus, hypothalamus and ventral striatum.

For quantification 10 µl of plasma or tissue homogenate was mixed with internal standards (D_4_-nicotinic acid, ^13^C_10_-kynurenine, D_4_-dopamine) and derivatized with phenylisothiocyanate for 1 h at room temperature. After drying under a stream of nitrogen the samples were extracted with methanol and diluted with water to 50%. Standard curves from known concentrations of commercial compounds were created. In addition to separate measurements, the blood plasma data was also pooled together from the second and third cohort to see more significant differences between the *Negr1-*deficient mice and the wild-type control mice.

### Statistical analysis

Data are presented as mean values ± standard error of the mean (SEM). Before the analyses, an outlier test was performed on all the data. Log-transformation was used to normalise the data before analysis. Normality of data distribution was assessed using the Shapiro–Wilk test. Brain metabolite levels were analysed using Student’s *t*-test or the Mann–Whitney *U* test for non-parametric data. Blood plasma metabolites and qPCR data were evaluated using two-way ANOVA followed by Tukey’s post hoc test. (In the supplementary, one-way ANOVA was used for blood plasma to allow pooling the data.)

Statistical analyses for behavioural experiments and metabolite measurements, as well as correlation plot generation, were conducted using R (version 4.3.1). Analysis of qPCR data and generation of all other graphs (excluding correlation plots) were performed using GraphPad Prism (version 10.2.1). Z-scores were calculated for each sample when necessary to standardise and compare data across groups (between different brain regions and blood serum) using the mean and standard deviation of the control group:

$$\:z=\frac{{x}_{i}-{\mu\:}_{control}}{{\sigma\:}_{control}}$$

where x_i_ is the log₂-transformed value for each subject, the group mean, and the standard deviation.

Statistical significance was defined as *p* < 0.05. Illustrative figures were created using BioRender.com.

## Supplementary Information

Below is the link to the electronic supplementary material.

Supplementary Material 1

## Data Availability

The data that support the findings of this study are available upon reasonable request to the corresponding author.
