# Supplementary material for: Negr1 deficiency alters glutamate signalling and kynurenine pathway in a mouse model of psychiatric disorders
Source: Sci Rep. 2026 Jan 16;16:5317. doi: 10.1038/s41598-026-35968-7 (PMC12881613; doi:10.1038/s41598-026-35968-7)
Supplement: Supplementary file 1 — Supplementary Material 1 [file 41598_2026_35968_MOESM1_ESM.docx]

**Supplementary**

***Negr1* Deficiency Alters Glutamate Signalling and Kynurenine Pathway in a Mouse Model of Psychiatric Disorders**

Carolin Kuuskmäe^1*^, Kaie Mikheim^1^, Narges Mohammadrahimi^1^, Kalle Kilk^2^, Maria Kaare^1^, Mohan Jayaram^1^, German Ilnitski^1^, Este Leidmaa^1^, Mari-Anne Philips^1^, Eero Vasar^1^

^1^Department of Physiology, Institute of Biomedicine and Translational Medicine, University of Tartu, Tartu, Estonia.

^2^Department of Biochemistry, Institute of Biomedicine and Translational Medicine, University of Tartu, Tartu, Estonia.

**
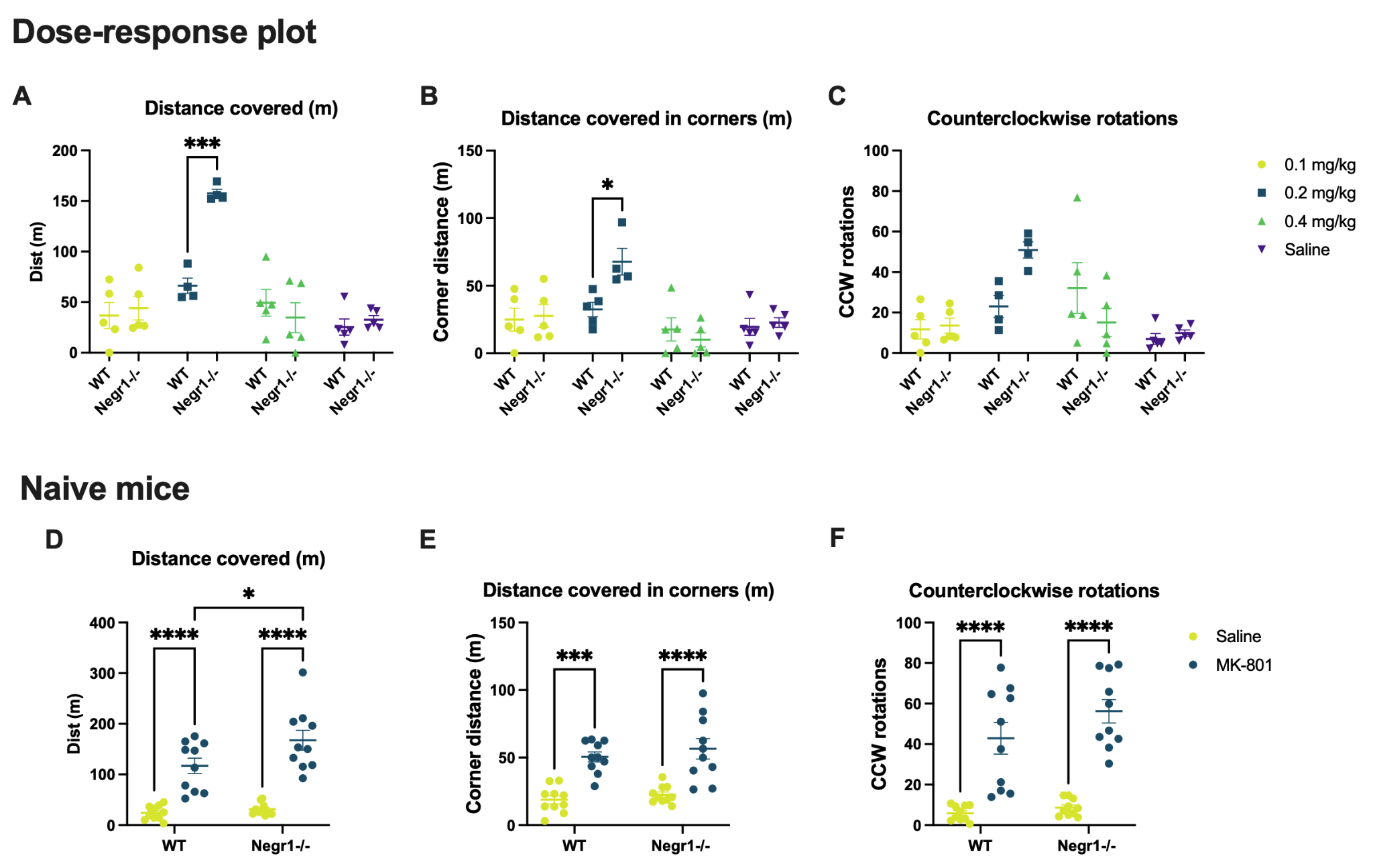
**

**Fig. S1. Dose–response effects of MK-801 (0.1–0.4 mg/kg) in wild-type (WT) and *Negr1*-deficient male mice, and the effect of MK-801 (0.2 mg/kg) in an independent cohort of drug-naive male mice from both genotypes.** The figure shows: (A) total distance covered, (B) distance covered in corners, and (C) number of counterclockwise rotations (n = 4–5 per group). Data are presented as mean ± SEM. Statistical analysis was performed using ordinary two-way ANOVA followed by Tukey's HSD test. *p < 0.05, ***p < 0.0001.

Based on these results, 0.2 mg/kg was selected as the most appropriate dose. (Note: Data for clockwise rotations were not usable; therefore, total rotation counts could not be calculated.)

(D) In a repeated MK-801 administration paradigm, *Negr1*-deficient male mice exhibited a blunted behavioural response over time. Because the mice in the repeated administration group had previously received a single MK-801 dose during the dose–response test (7 days prior), they were not fully drug-naive. We hypothesised that the heightened acute response to MK-801 is specific to drug-naive *Negr1*-deficient mice.

To test this, the acute administration experiment was repeated in an independent cohort of drug-naive male mice (n = 8–10, age 2.5 months). Supporting our hypothesis, *Negr1*-deficient mice in this new cohort again displayed an enhanced motor response, as indicated by increased total distance covered (p < 0.05).

**
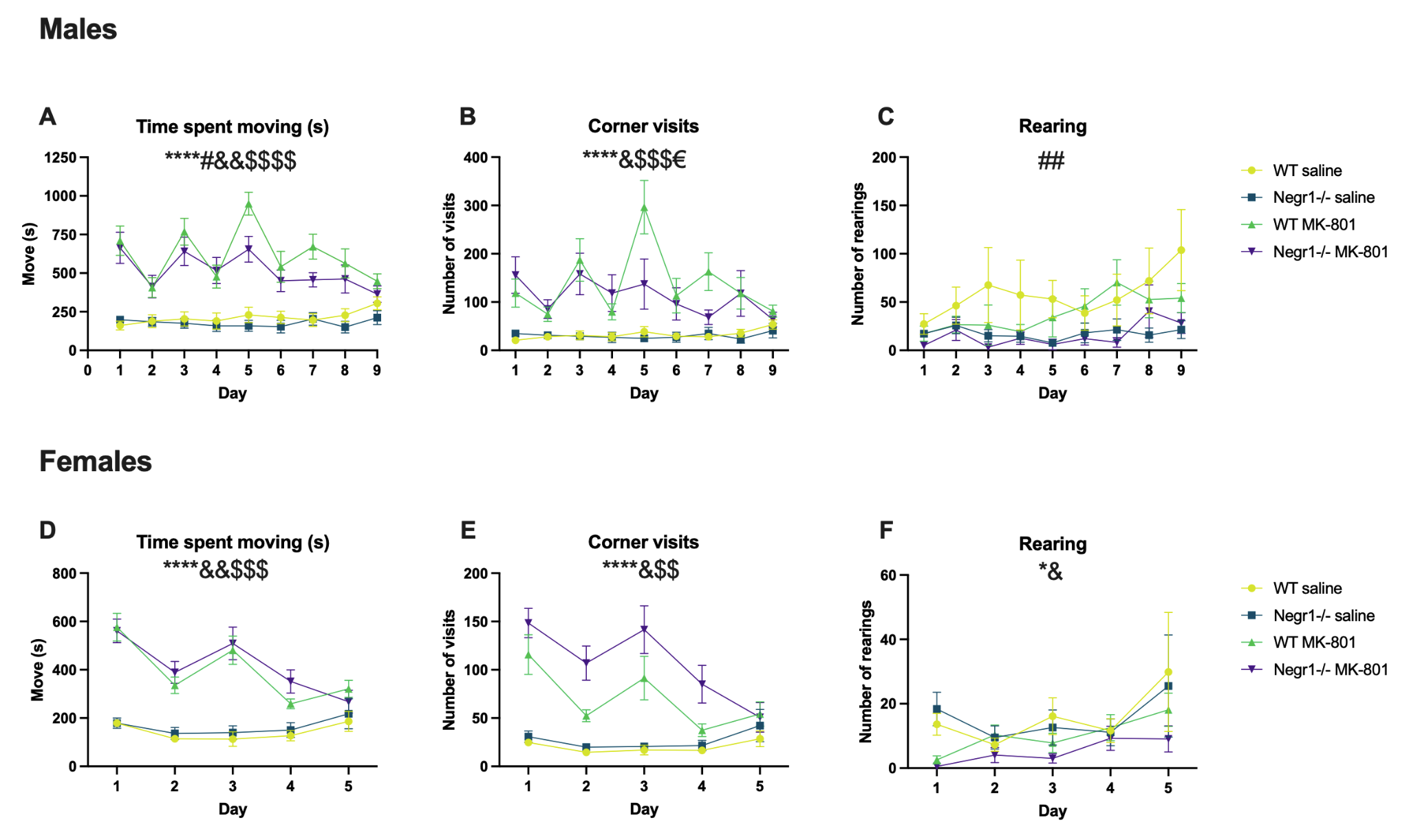
**

**Fig. S2. Effects of MK-801 on the behaviour of wild-type (WT) and *Negr1*-deficient mice.** The figure shows: (A, D) time spent moving, (B, E) number of corner visits, and (C, F) number of rearings in both male and female mice. Each dot represents the daily average per animal (males: *n* = 10; females: *n* = 8–16). Error bars indicate SEM. Main effects were analysed using three-way ANOVA followed by Tukey’s HSD test. Statistical significance is indicated by symbols above the graphs: * - treatment, # - genotype, & - day, $ - day and treatment interaction, € - genotype and treatment interaction. One symbol - p < 0.05, two symbols - p < 0.01, three symbols - p < 0.001, four symbols p < 0.0001.

**Effect of repeated treatment with MK-801 (0.2 mg/kg) on locomotor activity in male wild-type and *Negr1*-deficient mice (Fig. S1)**

**Day 1:** MK-801 significantly increased locomotor activity in both wild-type (WT) and *Negr1*^-/-^ mice versus saline controls. Time spent moving was elevated in both genotypes (WT: *p* < 0.0001; *Negr1*^-/-^: *p* < 0.001), as were corner visits (WT: *p* < 0.05; *Negr1*^-/-^: *p* < 0.01). No significant changes were observed in the number of rearings.

**Day 2:** A reduced locomotor response was observed following the second MK-801 administration. Nonetheless, time spent moving remained elevated in both WT and *Negr1*^-/-^ mice (*p* < 0.05). Increased corner visits were observed only in *Negr1*^-/-^ mice (*p* < 0.05). The number of rearings remained unchanged.

**Day 3:** MK-801 again induced strong stimulation, particularly in WT mice. Time spent moving increased significantly (WT: *p* < 0.00001; *Negr1*^-/-^: *p* < 0.001), as did corner visits (WT: *p* < 0.01; *Negr1*^-/-^: *p* < 0.05).

**Day 4:** Stimulatory effects began to decline. Time spent moving was modestly elevated (WT: *p* < 0.05; *Negr1*^-/-^: *p* < 0.01), with increased corner observed only in *Negr1^-/-^* mice (*p* < 0.05). Rearings continued to show no significant differences.

**Day 5:** WT mice exhibited peak locomotor activity (time spent moving: *p* < 1×10⁻⁷; corner visits: *p* < 0.001), while *Negr1^-/-^* mice showed no further increase or even reductions in activity, resulting in significant genotype differences in both time spent moving and corner visits (*p* < 0.05). Rearings remained unaffected.

**Day 6:** MK-801 effects diminished further. WT mice showed reduced locomotor activity and fewer corner entries; *Negr1^-/-^* mice exhibited an additional decline, furthering Day 2 levels.

**Days 7–9:** Continued MK-801 administration led to a progressive attenuation of locomotor effects. By Day 9, activity levels had significantly declined in both genotypes compared to Days 3 and 5. In contrast, rearing behaviour appeared to increase slightly but did not reach statistical significance.

**Effect of Repeated Treatment with MK-801 (0.2 mg/kg) on Locomotor Activity in Female Wild-Type and *Negr1*-Deficient Mice (Fig. S1)**

**Day 1**: Similar to males, MK-801 significantly increased locomotor activity in both wild-type (WT) and *Negr1^-/-^* females (*p* < 0.001) along with elevated corner visits (WT: *p* < 0.05; *Negr1^-/-^*: *p* < 0.01). In contrast to males, rearings also increased significantly (WT: *p* < 0.05; *Negr1^-/-^*: *p* < 0.0001).

**Day 2**: A reduced response was observed, although time spent moving remained elevated in both genotypes (*p* < 0.05). Increased corner visits were noted only in *Negr1^-/-^* mice (*p* < 0.01). Rearings showed no significant change.

**Day 3**: Unlike in males, MK-801 did not fully restore the initial stimulatory effect in females. Locomotion remained modestly elevated (WT and *Negr1^-/-^*: *p* < 0.05), but only *Negr1^-/-^* mice showed increased corner visits (*p* < 0.05). The number of rearings remained unchanged.

**Days 4 and 5**: Both locomotion and corner visits declined to near saline-treated control levels, with a marked reduction compared to Day 1. Rearings showed a slight, non-significant increase. The diminishing behavioural response indicated the development of tolerance to MK-801, leading to termination of treatment in females after Day 5.

**Gender Differences in Response to MK-801**

By Day 5, clear sex-dependent differences in behavioural responses emerged, prompting direct comparisons with Day 1.

**Time spent in locomotion:** On Day 5, wild-type males showed increased locomotion compared to Day 1 (*p* < 0.05), unlike *Negr1^-/-^* males. WT males also displayed greater activity than *Negr1^-/-^* males (*p* < 0.05). In contrast, both WT and *Negr1^-/-^* females showed significant reduction in locomotion compared to Day 1 (WT: *p* < 0.01; *Negr1^-/-^:* *p* < 0.0001), and their activity levels were significantly lower than those of their male counterparts (WT: *p* < 1×10⁻⁵; *Negr1^-/-^*: *p* < 0.01).

**Corner visits:** WT males exhibited increased corner visits on Day 5 (*p* < 0.05), while *Negr1^-/-^* males showed no change. Among females, *Negr1^-/-^* mice showed a significant reduction in corner visits (*p* < 0.001), and WT females showed a milder reduction (*p* < 0.05). On Day 5, WT female mice responded less than WT males (*p* < 0.01), while no sex difference was observed in the *Negr1^-/-^* group.

**Rearings:** *Negr1^-/-^* males treated with saline had fewer rearings on Day 5 compared to Day 1 (*p* < 0.01); other male groups showed no significant changes. Among females, WT mice treated with MK-801 exhibited increased rearings (*p* < 0.01). No other significant effects on rearing behaviour were observed.


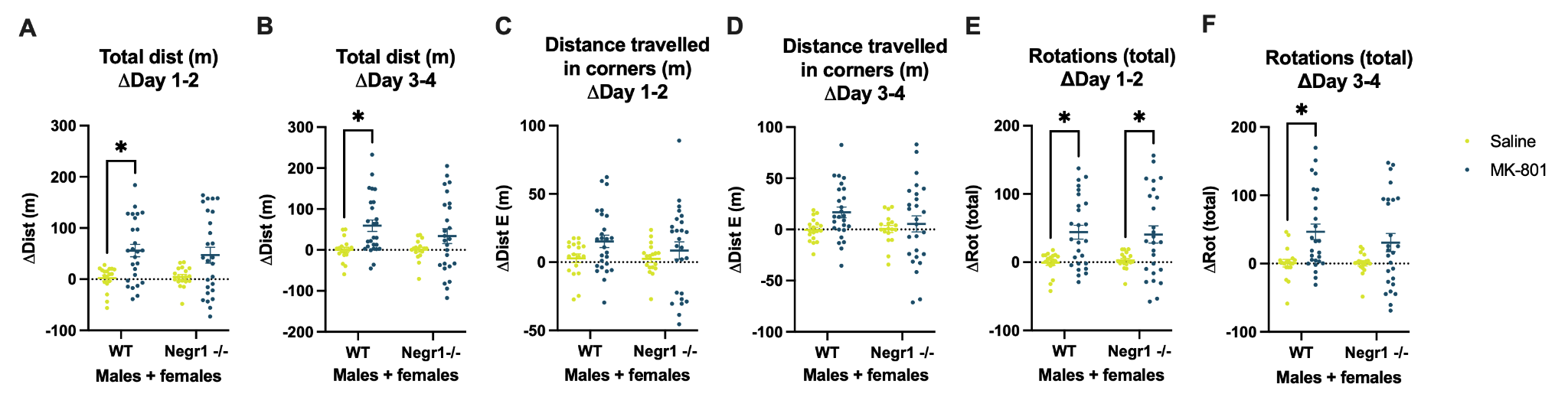


**Fig. S3. Blunted progression of rapid behavioural tolerance during every-other-day MK-801 administration in *Negr1*-deficient mice.** MK-801-induced locomotor activity and stereotypic behaviour progressively declined on alternating days during chronic treatment in both male and female mice. Delta values for Days 1–2 (A, C, E) and Days 3–4 (B, D, F) represent changes in activity between each pair of treatment days. Compared to wild-type (WT) controls, *Negr1^-/-^* mice showed smaller reductions in behavioural responses, indicating a blunted progression of rapid behavioural tolerance. These genotype-dependent differences suggest altered NMDA receptor sensitivity in *Negr1^-/-^* mice.

**Table S1. Three-way ANOVA results for MK-801 effect on wild-type (WT) and *Negr1*-deficient mice’s behaviour.**

| Total distance covered (m) - males | ANOVA table | DF | F (DFn, DFd) | P value |
| --- | --- | --- | --- | --- |
|  | Day | 8 | F (2.694, 96.99) = 5.835 | P=0.0016 |
|  | **Treatment** | **1** | **F (1, 36) = 138.4** | **P<0.0001** |
|  | Genotype | 1 | F (1, 36) = 5.023 | P=0.0313 |
|  | **Day x Treatment** | **8** | **F (8, 288) = 7.000** | **P<0.0001** |
|  | Day x Genotype | 8 | F (8, 288) = 1.530 | P=0.1462 |
|  | Treatment  x Genotype | 1 | F (1, 36) = 2.234 | P=0.1437 |
|  | Day x Treatment  x Genotype | 8 | F (8, 288) = 1.180 | P=0.3106 |
| Total distance covered (m) - females | Day | 4 | F (1.796, 79.04) = 6.528 | P=0.0033 |
|  | **Treatment** | **1** | **F (1, 44) = 46.94** | **P<0.0001** |
|  | Genotype | 1 | F (1, 44) = 0.5909 | P=0.4462 |
|  | **Day x Treatment** | **4** | **F (4, 176) = 7.053** | **P<0.0001** |
|  | Day x Genotype | 4 | F (4, 176) = 0.2328 | P=0.9196 |
|  | Treatment  x Genotype | 1 | F (1, 44) = 0.08840 | P=0.7676 |
|  | Day x Treatment  x Genotype | 4 | F (4, 176) = 0.3140 | P=0.8684 |
| Distance covered in corners (m) - males | Day | 8 | F (2.465, 88.73) = 1.069 | P=0.3579 |
|  | **Treatment** | **1** | **F (1, 36) = 106.9** | **P<0.0001** |
|  | Genotype | 1 | F (1, 36) = 4.963 | P=0.0322 |
|  | Day x Treatment | 8 | F (8, 288) = 1.798 | P=0.0772 |
|  | Day  x Genotype | 8 | F (8, 288) = 0.8253 | P=0.5808 |
|  | Treatment  x Genotype | 1 | F (1, 36) = 4.178 | P=0.0483 |
|  | Day x Treatment  x Genotype | 8 | F (8, 288) = 1.122 | P=0.3485 |
| Distance covered in corners (m) - females | Day | 4 | F (2.452, 107.9) = 3.776 | P=0.0187 |
|  | **Treatment** | **1** | **F (1, 44) = 54.54** | **P<0.0001** |
|  | Genotype | 1 | F (1, 44) = 0.02133 | P=0.8845 |
|  | Day x Treatment | 4 | F (4, 176) = 3.662 | P=0.0068 |
|  | Day x Genotype | 4 | F (4, 176) = 0.6053 | P=0.6594 |
|  | Treatment  x Genotype | 1 | F (1, 44) = 0.06169 | P=0.8050 |
|  | Day x Treatment  x Genotype | 4 | F (4, 176) = 0.5096 | P=0.7288 |
| Total rotations made - males | **Day** | **8** | **F (3.102, 111.7) = 8.656** | **P<0.0001** |
|  | **Treatment** | **1** | **F (1, 36) = 146.8** | **P<0.0001** |
|  | Genotype | 1 | F (1, 36) = 4.594 | P=0.0389 |
|  | **Day x Treatment** | **8** | **F (8, 288) = 9.988** | **P<0.0001** |
|  | Day x Genotype | 8 | F (8, 288) = 1.205 | P=0.2955 |
|  | Treatment  x Genotype | 1 | F (1, 36) = 0.6735 | P=0.4172 |
|  | Day x Treatment  x Genotype | 8 | F (8, 288) = 0.8226 | P=0.5832 |
| Total rotations made - females | Day | 4 | F (1.989, 87.52) = 6.128 | P=0.0033 |
|  | **Treatment** | **1** | **F (1, 44) = 38.75** | **P<0.0001** |
|  | Genotype | 1 | F (1, 44) = 0.9073 | P=0.3460 |
|  | **Day x Treatment** | **4** | **F (4, 176) = 6.800** | **P<0.0001** |
|  | Day x Genotype | 4 | F (4, 176) = 0.1165 | P=0.9765 |
|  | Treatment  x Genotype | 1 | F (1, 44) = 0.2287 | P=0.6349 |
|  | Day x Treatment  x Genotype | 4 | F (4, 176) = 0.2100 | P=0.9326 |
| Time spent moving - males | Day | 8 | F (2.197, 79.09) = 4.840 | P=0.0085 |
|  | **Treatment** | **1** | **F (1, 36) = 165.5** | **P<0.0001** |
|  | Genotype | 1 | F (1, 36) = 5.680 | P=0.0226 |
|  | **Day x Treatment** | **8** | **F (8, 288) = 6.114** | **P<0.0001** |
|  | Day x Genotype | 8 | F (8, 288) = 1.026 | P=0.4162 |
|  | Treatment  x Genotype | 1 | F (1, 36) = 1.310 | P=0.2600 |
|  | Day x Treatment  x Genotype | 8 | F (8, 288) = 0.7307 | P=0.6643 |
| Time spent moving - females | Day | 4 | F (2.003, 88.15) = 5.935 | P=0.0038 |
|  | **Treatment** | **1** | **F (1, 44) = 43.82** | **P<0.0001** |
|  | Genotype | 1 | F (1, 44) = 0.2711 | P=0.6052 |
|  | **Day x Treatment** | **4** | **F (4, 176) = 5.963** | **P=0.0002** |
|  | Day x Genotype | 4 | F (4, 176) = 0.2666 | P=0.8991 |
|  | Treatment  x Genotype | 1 | F (1, 44) = 0.001680 | P=0.9675 |
|  | Day x Treatment  x Genotype | 4 | F (4, 176) = 0.2989 | P=0.8784 |
| Corner visits - males | Day | 8 | F (3.360, 121.0) = 3.456 | P=0.0150 |
|  | **Treatment** | **1** | **F (1, 36) = 59.92** | **P<0.0001** |
|  | Genotype | 1 | F (1, 36) = 1.351 | P=0.2528 |
|  | **Day x Treatment** | **8** | **F (8, 288) = 4.028** | **P=0.0001** |
|  | Day x Genotype | 8 | F (8, 288) = 2.036 | P=0.0423 |
|  | Treatment  x Genotype | 1 | F (1, 36) = 0.9624 | P=0.3331 |
|  | Day x Treatment  x Genotype | 8 | F (8, 288) = 1.623 | P=0.1178 |
| Corner visits - females | Day | 4 | F (1.840, 80.97) = 3.451 | P=0.0401 |
|  | **Treatment** | **1** | **F (1, 44) = 24.53** | **P<0.0001** |
|  | Genotype | 1 | F (1, 44) = 2.020 | P=0.1623 |
|  | Day x Treatment | 4 | F (4, 176) = 4.059 | P=0.0036 |
|  | Day x Genotype | 4 | F (4, 176) = 0.2445 | P=0.9127 |
|  | Treatment  x Genotype | 1 | F (1, 44) = 0.8043 | P=0.3747 |
|  | Day x Treatment  x Genotype | 4 | F (4, 176) = 0.4590 | P=0.7658 |
| Rearing - males | Day | 8 | F (1.918, 69.05) = 1.786 | P=0.1767 |
|  | Treatment | 1 | F (1, 36) = 1.234 | P=0.2739 |
|  | Genotype | 1 | F (1, 36) = 10.92 | P=0.0022 |
|  | Day x Treatment | 8 | F (8, 288) = 0.4400 | P=0.8964 |
|  | Day x Genotype | 8 | F (8, 288) = 0.7590 | P=0.6393 |
|  | Treatment  x Genotype | 1 | F (1, 36) = 0.7762 | P=0.3841 |
|  | Day x Treatment  x Genotype | 8 | F (8, 288) = 0.7576 | P=0.6405 |
| Rearing - females | Day | 4 | F (2.015, 88.68) = 4.329 | P=0.0158 |
|  | Treatment | 1 | F (1, 44) = 4.616 | P=0.0372 |
|  | Genotype | 1 | F (1, 44) = 0.4215 | P=0.5196 |
|  | Day x Treatment | 4 | F (4, 176) = 1.966 | P=0.1017 |
|  | Day x Genotype | 4 | F (4, 176) = 0.4282 | P=0.7882 |
|  | Treatment  x Genotype | 1 | F (1, 44) = 0.3252 | P=0.5714 |
|  | Day x Treatment  x Genotype | 4 | F (4, 176) = 0.1625 | P=0.9570 |


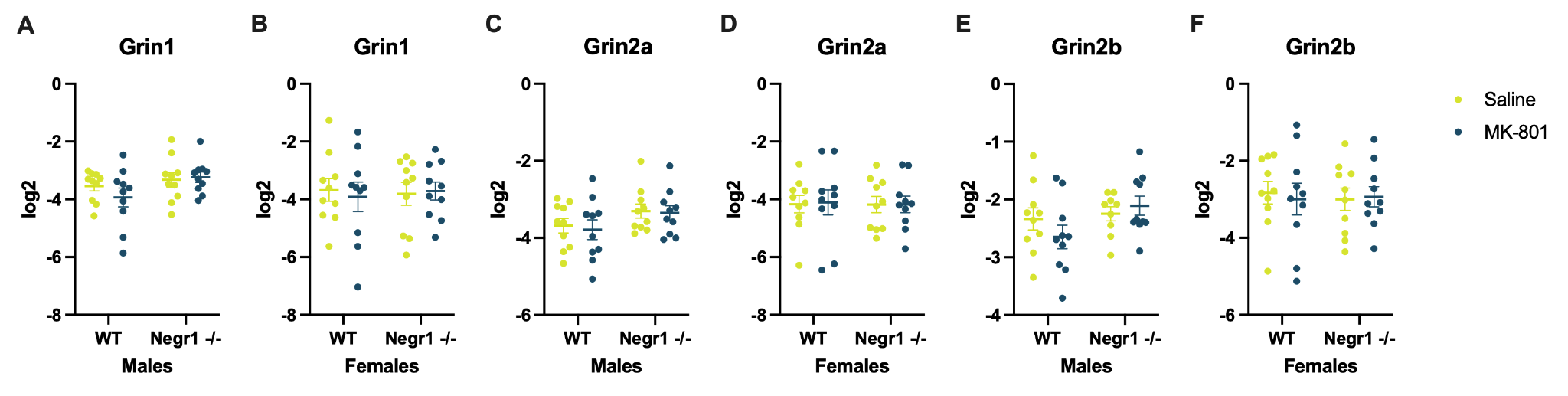


**Fig. S4. NMDA receptor-related gene expression in the ventral striatum of mice.** The figure shows gene expression of *Grin1* (A, B), *Grin2a* (C, D), *Grin2b* (E, F) in both male and female mice. Each graph includes four groups: WT mice injected with physiological solution (saline), WT mice injected with MK-801, *Negr1*-deficient mice injected with physiological solution, and *Negr1*-deficient mice injected with MK-801. No statistically significant differences were found between the four groups. However, a significant sex difference was found in the expression of *Grin2a* (F1,72 = 10.40, p < 0.01), *Grin2b* (F1,72 = 11.99, p < 0.001). Each group consisted of 8–10 mice. Data are presented as mean ± SEM. Statistical analysis was conducted using ordinary two-way ANOVA followed by Tukey’s HSD test. WT – wild-type.


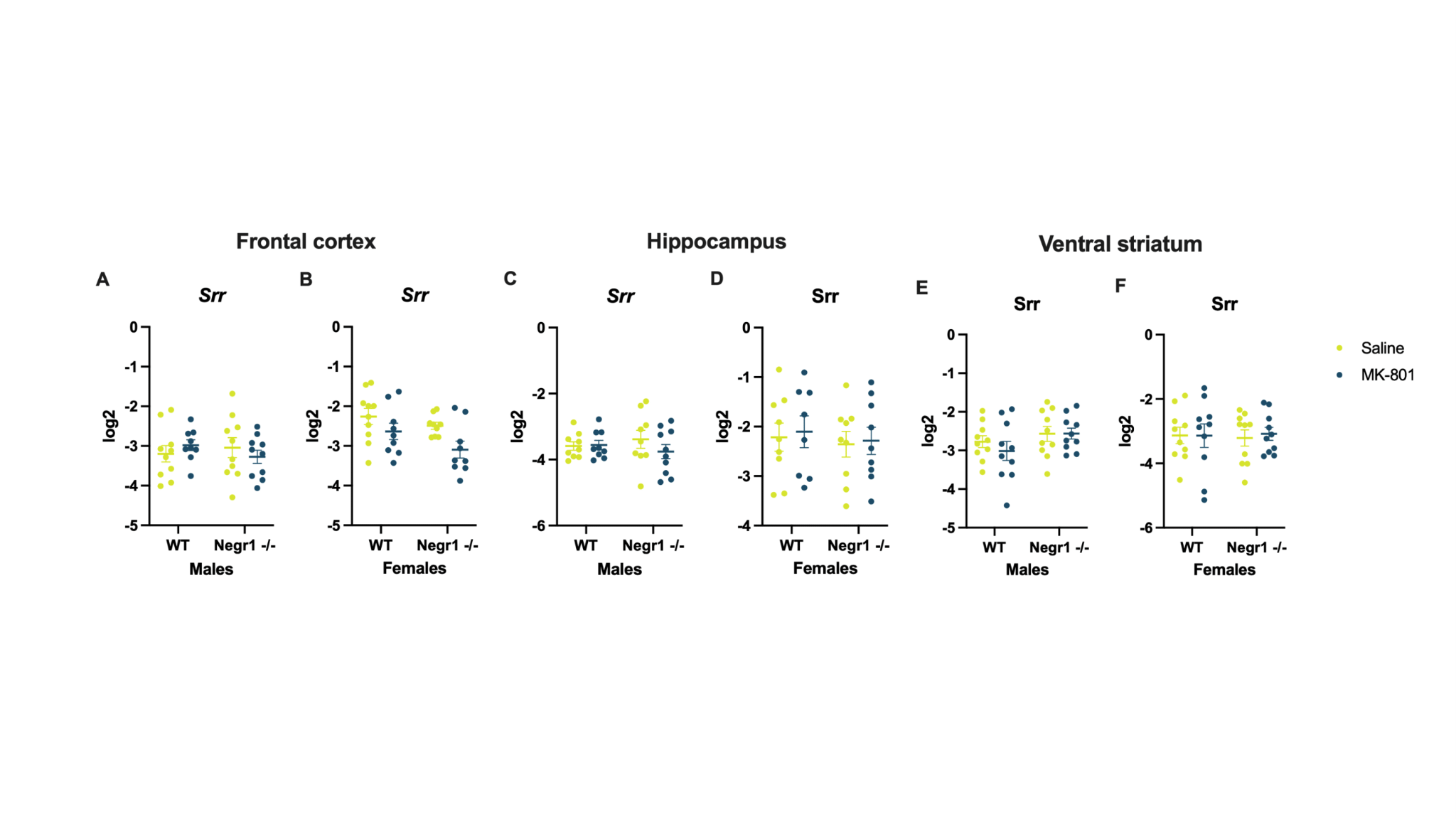


**Fig. S5. *Srr* expression in different regions of the mouse brain.** The figure shows *Srr* gene expression in both male and female mice across three brain regions: frontal cortex (A, B), hippocampus (C, D), and ventral striatum (E, F). Each graph includes four groups: wild-type (WT) mice injected with saline, WT mice injected with MK-801, *Negr1*-deficient mice injected with saline, and *Negr1*-deficient mice injected with MK-801. No statistically significant differences were observed between the four groups in any of the brain regions. Each group consisted of 8–10 mice. Data are presented as mean ± SEM. Statistical analysis was performed using ordinary two-way ANOVA followed by Tukey’s HSD test.

**
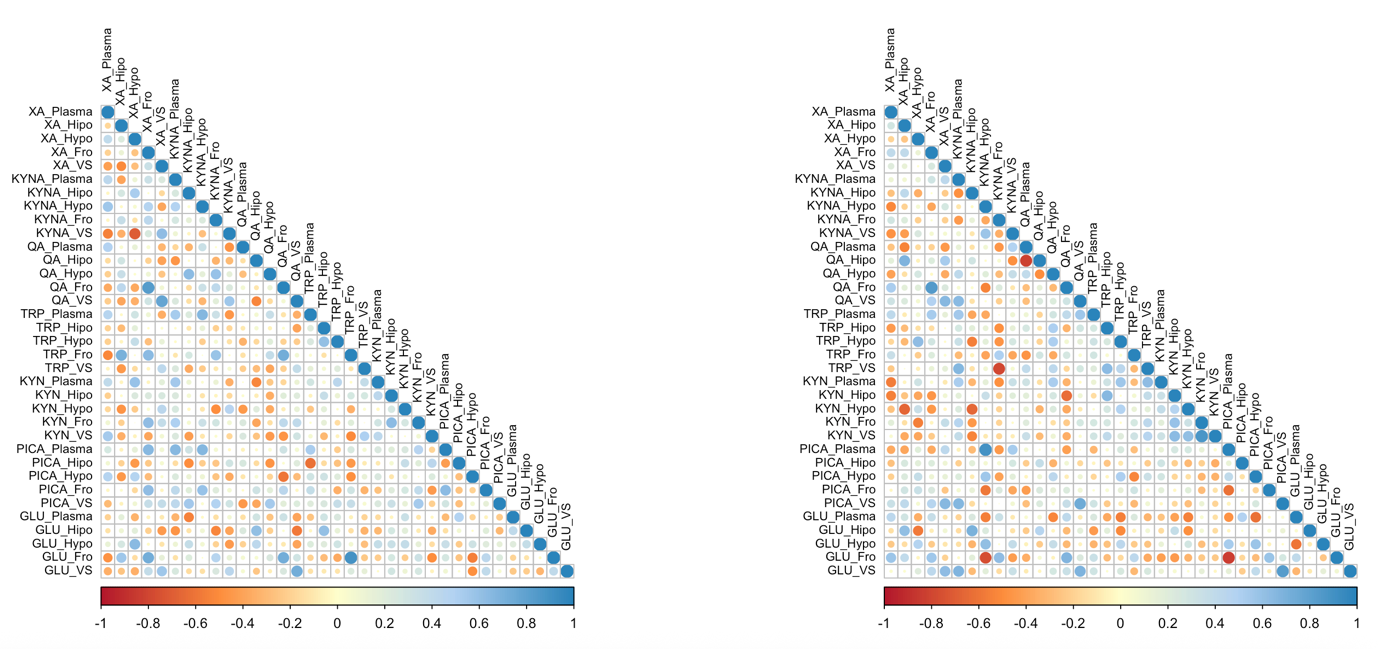
**

**Fig. S6. Correlations among kynurenine pathway metabolites, tryptophan, and glutamate in male mice.** Data are shown for five regions: frontal cortex (Fro), hippocampus (Hipo), hypothalamus (Hypo), ventral striatum (VS), and blood plasma (Plasma). Seven metabolites relevant to the study were included in the correlation analysis: KYNA – kynurenic acid, KYN – kynurenine, TRP – tryptophan, QA – quinolinic acid, PICA – picolinic acid, XA – xanthurenic acid, and GLU – glutamate. Correlation matrices are presented for wild-type mice (left) and *Negr1^-/-^* mice (right).

**
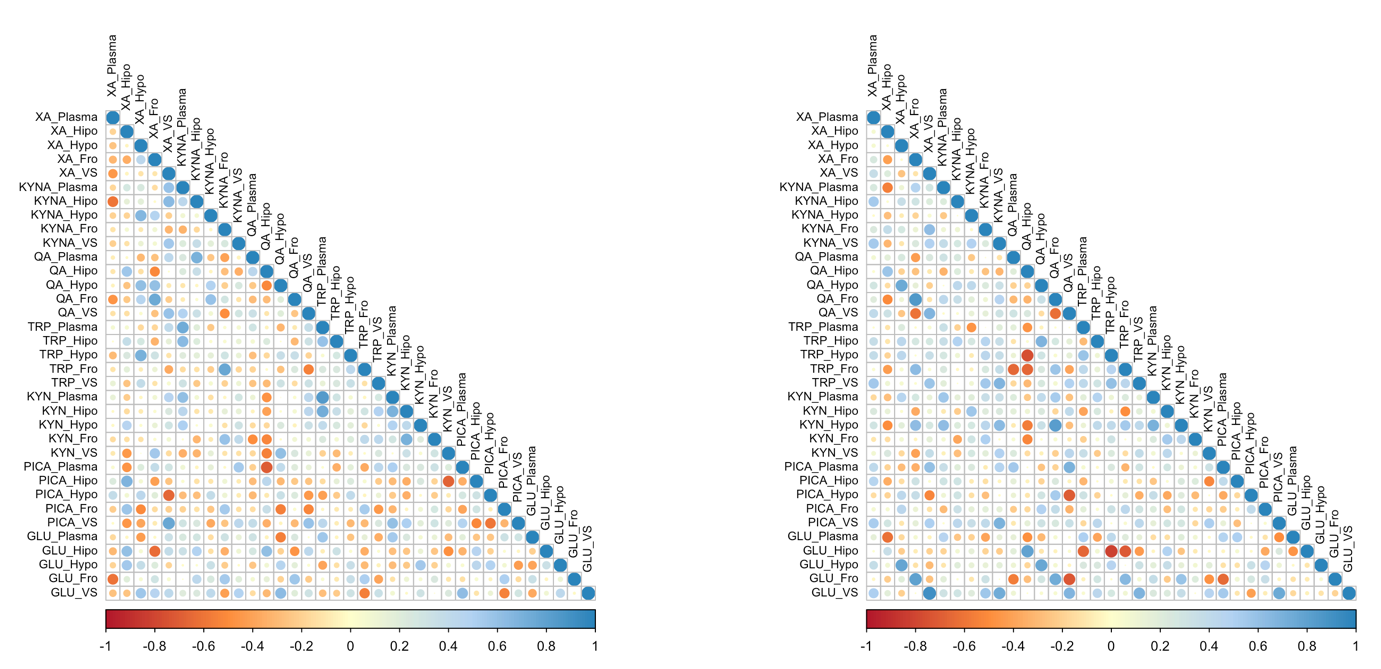
**

**Fig. S7. Correlations among kynurenine pathway metabolites, tryptophan, and glutamate in female mice.** Data are shown for five regions: frontal cortex (Fro), hippocampus (Hipo), hypothalamus (Hypo), ventral striatum (VS), and blood plasma (Plasma). Seven metabolites most relevant to the study were included in the correlation analysis: KYNA – kynurenic acid, KYN – kynurenine, TRP – tryptophan, QA – quinolinic acid, PICA – picolinic acid, XA – xanthurenic acid, and GLU – glutamate. Correlation matrices are presented for wild-type mice (left) and *Negr1^-/-^* mice (right).

**Table S2. Significant correlations among kynurenine pathway metabolites, tryptophan, and glutamate in male wild-type (WT) mice.** Correlation data are presented for five regions: frontal cortex (Fro), hippocampus (Hipo), hypothalamus (Hypo), ventral striatum (VS), and blood plasma (Plasma). Seven metabolites most relevant to the study were included in the analysis: KYNA – kynurenic acid, KYN – kynurenine, TRP – tryptophan, QA – quinolinic acid, PICA – picolinic acid, XA – xanthurenic acid, and GLU – glutamate. Correlation = r-value.

| **Variable_1** | **Variable_2** | **Correlation** | **P_Value** |
| --- | --- | --- | --- |
| KYNA_Hypo | XA_Plasma | 0.566958 | 0.034496 |
| KYNA_VS | XA_Plasma | -0.54248 | 0.045043 |
| KYN_VS | XA_Plasma | 0.549295 | 0.041897 |
| TRP_Fro | XA_Hipo | 0.701554 | 0.00517 |
| GLU_Fro | XA_Hipo | 0.568364 | 0.033952 |
| KYNA_VS | XA_Hypo | -0.70716 | 0.004677 |
| KYN_Plasma | XA_Hypo | 0.597542 | 0.024031 |
| **Variable_1** | **Variable_2** | **Correlation** | **P_Value** |
| GLU_Hypo | XA_Hypo | 0.641085 | 0.013485 |
| QA_Fro | XA_Fro | 0.837661 | 0.000184 |
| TRP_Fro | XA_Fro | 0.648712 | 0.012081 |
| KYN_Fro | XA_Fro | 0.626565 | 0.016499 |
| PICA_Plasma | XA_Fro | 0.658443 | 0.010455 |
| PICA_Fro | XA_Fro | 0.623856 | 0.017114 |
| GLU_Fro | XA_Fro | 0.723132 | 0.003472 |
| KYNA_VS | XA_VS | 0.628992 | 0.015963 |
| QA_VS | XA_VS | 0.779777 | 0.001006 |
| GLU_VS | XA_VS | 0.566213 | 0.034787 |
| KYN_Plasma | KYNA_Plasma | 0.550631 | 0.0413 |
| PICA_Plasma | KYNA_Plasma | 0.655356 | 0.010951 |
| QA_Hypo | KYNA_Hipo | 0.631185 | 0.015489 |
| PICA_VS | KYNA_Hipo | 0.556023 | 0.038952 |
| GLU_Plasma | KYNA_Hipo | -0.54344 | 0.044588 |
| TRP_Plasma | KYNA_Hypo | 0.664055 | 0.009598 |
| PICA_Plasma | KYNA_Hypo | 0.663077 | 0.009743 |
| PICA_Fro | KYNA_Hypo | 0.597594 | 0.024016 |
| QA_Hypo | KYNA_Fro | 0.595404 | 0.024673 |
| TRP_Fro | KYNA_Fro | 0.584178 | 0.028259 |
| QA_VS | KYNA_VS | 0.561633 | 0.036616 |
| GLU_Hipo | QA_Hipo | 0.617638 | 0.018591 |
| PICA_VS | QA_Hypo | 0.542745 | 0.044917 |
| TRP_Fro | QA_Fro | 0.721911 | 0.003554 |
| PICA_Hypo | QA_Fro | -0.59197 | 0.025733 |
| GLU_Fro | QA_Fro | 0.726629 | 0.003244 |
| GLU_Hipo | QA_VS | -0.5701 | 0.033288 |
| GLU_VS | QA_VS | 0.724612 | 0.003374 |
| PICA_Hipo | TRP_Plasma | -0.61682 | 0.018791 |
| TRP_Hypo | TRP_Hipo | 0.602871 | 0.022485 |
| GLU_Hipo | TRP_Hipo | 0.607966 | 0.021078 |
| GLU_Fro | TRP_Fro | 0.893497 | 1.67E-05 |
| KYN_Fro | KYN_Hipo | 0.630437 | 0.01565 |
| PICA_VS | KYN_Fro | 0.545619 | 0.043573 |
| PICA_Fro | PICA_Plasma | 0.637615 | 0.014163 |
| GLU_Fro | PICA_Hypo | -0.55681 | 0.03862 |

**Table S3. Significant correlations among kynurenine pathway metabolites, tryptophan, and glutamate in male *Negr1^-/-^* mice.** Correlation data are presented for five regions: frontal cortex (Fro), hippocampus (Hipo), hypothalamus (Hypo), ventral striatum (VS), and blood plasma (Plasma). Seven metabolites most relevant to the study were included in the analysis: KYNA – kynurenic acid, KYN – kynurenine, TRP – tryptophan, QA – quinolinic acid, PICA – picolinic acid, XA – xanthurenic acid, and GLU – glutamate. Correlation = r-value.

| **Variable_1** | **Variable_2** | **Correlation** | **P_Value** |
| --- | --- | --- | --- |
| KYN_Plasma | XA_Plasma | -0.56162 | 0.045787 |
| GLU_Fro | XA_Plasma | 0.573069 | 0.04063 |
| QA_Hipo | XA_Hipo | 0.684953 | 0.009785 |
| KYN_Hypo | XA_Hipo | -0.66581 | 0.012985 |
| GLU_Hipo | XA_Hipo | 0.61687 | 0.024719 |
| **Variable_1** | **Variable_2** | **Correlation** | **P_Value** |
| TRP_Hypo | XA_Hypo | 0.629257 | 0.021206 |
| KYN_Fro | XA_Hypo | -0.55728 | 0.047863 |
| GLU_Hipo | XA_Hypo | -0.57502 | 0.039795 |
| QA_Fro | XA_Fro | 0.841471 | 0.000311 |
| GLU_Fro | XA_Fro | 0.569263 | 0.042296 |
| QA_VS | XA_VS | 0.651838 | 0.015775 |
| PICA_VS | XA_VS | 0.657478 | 0.014599 |
| GLU_VS | XA_VS | 0.624851 | 0.02241 |
| QA_VS | KYNA_Plasma | 0.648407 | 0.016524 |
| TRP_VS | KYNA_Plasma | 0.664163 | 0.013292 |
| PICA_VS | KYNA_Plasma | 0.668468 | 0.012498 |
| GLU_VS | KYNA_Plasma | 0.673262 | 0.011657 |
| KYN_Hypo | KYNA_Hipo | -0.65432 | 0.015248 |
| GLU_Hipo | KYNA_Hipo | 0.69217 | 0.008749 |
| KYNA_VS | KYNA_Hypo | 0.570911 | 0.041569 |
| PICA_Plasma | KYNA_Hypo | 0.896552 | 3.32E-05 |
| PICA_Hypo | KYNA_Hypo | 0.569698 | 0.042103 |
| PICA_Fro | KYNA_Hypo | -0.58448 | 0.035922 |
| GLU_Plasma | KYNA_Hypo | -0.5546 | 0.049176 |
| GLU_Hypo | KYNA_Hypo | 0.612295 | 0.026119 |
| GLU_Fro | KYNA_Hypo | -0.76984 | 0.002084 |
| TRP_VS | KYNA_Fro | -0.79494 | 0.001163 |
| GLU_Fro | KYNA_Fro | 0.624224 | 0.022585 |
| QA_Hipo | QA_Plasma | -0.81836 | 0.000627 |
| KYN_Hipo | QA_Fro | -0.63968 | 0.018551 |
| GLU_Fro | QA_Fro | 0.679601 | 0.010611 |
| TRP_Plasma | QA_VS | 0.56108 | 0.046043 |
| PICA_VS | QA_VS | 0.743177 | 0.003602 |
| GLU_VS | QA_VS | 0.684694 | 0.009824 |
| TRP_VS | TRP_Hipo | 0.632578 | 0.020331 |
| KYN_Hipo | TRP_Hipo | 0.65742 | 0.014611 |
| KYN_Plasma | TRP_Hypo | 0.569741 | 0.042084 |
| KYN_Plasma | TRP_VS | 0.621527 | 0.023351 |
| KYN_Fro | KYN_Hipo | 0.594963 | 0.031957 |
| KYN_VS | KYN_Hipo | 0.636276 | 0.019389 |
| KYN_VS | KYN_Hypo | 0.61585 | 0.025026 |
| KYN_VS | KYN_Fro | 0.879846 | 7.31E-05 |
| PICA_Fro | PICA_Plasma | -0.61065 | 0.026638 |
| GLU_Fro | PICA_Plasma | -0.83904 | 0.000336 |
| GLU_Plasma | PICA_Hypo | -0.61978 | 0.023858 |
| GLU_Hypo | PICA_Hypo | 0.573868 | 0.040287 |
| GLU_Fro | PICA_Fro | 0.580901 | 0.037353 |
| GLU_VS | PICA_VS | 0.832352 | 0.000415 |
| GLU_Hypo | GLU_Plasma | -0.59831 | 0.030763 |

**Table S4. Significant correlations among kynurenine pathway metabolites, tryptophan, and glutamate in female wild-type (WT) mice.** Correlation data are presented for five regions: frontal cortex (Fro), hippocampus (Hipo), hypothalamus (Hypo), ventral striatum (VS), and blood plasma (Plasma). Seven metabolites most relevant to the study were included in the analysis: KYNA – kynurenic acid, KYN – kynurenine, TRP – tryptophan, QA – quinolinic acid, PICA – picolinic acid, XA – xanthurenic acid, and GLU – glutamate. Correlation = r-value.

| **Variable_1** | **Variable_2** | **Correlation** | **P_Value** |
| --- | --- | --- | --- |
| KYNA_Hipo | XA_Plasma | -0.59545 | 0.02466 |
| **Variable_1** | **Variable_2** | **Correlation** | **P_Value** |
| GLU_Fro | XA_Plasma | -0.60368 | 0.022258 |
| QA_Hipo | XA_Hipo | 0.560746 | 0.036979 |
| PICA_Hipo | XA_Hipo | 0.684782 | 0.006891 |
| PICA_Fro | XA_Hipo | 0.577817 | 0.030456 |
| GLU_Hipo | XA_Hipo | 0.602913 | 0.022473 |
| GLU_Hypo | XA_Hipo | 0.550251 | 0.041469 |
| KYNA_Hypo | XA_Hypo | 0.667121 | 0.009153 |
| QA_Hypo | XA_Hypo | 0.613144 | 0.019717 |
| TRP_Hypo | XA_Hypo | 0.705713 | 0.004801 |
| GLU_VS | XA_Hypo | 0.560349 | 0.037142 |
| QA_Hypo | XA_Fro | 0.603659 | 0.022263 |
| QA_Fro | XA_Fro | 0.75355 | 0.001857 |
| KYN_VS | XA_Fro | 0.550192 | 0.041495 |
| GLU_Hipo | XA_Fro | -0.63794 | 0.014098 |
| KYNA_Plasma | XA_VS | 0.59494 | 0.024814 |
| KYNA_Hipo | XA_VS | 0.648778 | 0.012069 |
| KYNA_VS | XA_VS | 0.558918 | 0.037734 |
| QA_VS | XA_VS | 0.593734 | 0.025184 |
| PICA_Hypo | XA_VS | -0.66143 | 0.009992 |
| PICA_VS | XA_VS | 0.769368 | 0.001295 |
| TRP_Plasma | KYNA_Plasma | 0.73373 | 0.002817 |
| TRP_Hipo | KYNA_Plasma | 0.645006 | 0.012748 |
| KYN_Plasma | KYNA_Plasma | 0.61528 | 0.019176 |
| QA_Plasma | KYNA_Hipo | 0.705316 | 0.004835 |
| QA_Fro | KYNA_Hypo | 0.593978 | 0.025109 |
| TRP_Fro | KYNA_Fro | 0.768838 | 0.001311 |
| KYN_Fro | KYNA_Fro | 0.597736 | 0.023973 |
| PICA_Fro | KYNA_Fro | 0.546214 | 0.043298 |
| GLU_Fro | KYNA_Fro | 0.546362 | 0.04323 |
| PICA_Plasma | KYNA_VS | 0.549107 | 0.041981 |
| KYN_VS | QA_Hipo | -0.54628 | 0.04327 |
| PICA_Plasma | QA_Hipo | -0.70344 | 0.005 |
| GLU_Hipo | QA_Hipo | 0.613847 | 0.019538 |
| KYN_VS | QA_Hypo | 0.612797 | 0.019806 |
| PICA_Fro | QA_Hypo | -0.55046 | 0.041377 |
| GLU_Plasma | QA_Hypo | -0.55051 | 0.041353 |
| GLU_VS | QA_Hypo | 0.638113 | 0.014064 |
| GLU_Fro | QA_Fro | 0.567442 | 0.034308 |
| TRP_Fro | QA_VS | -0.54414 | 0.044262 |
| PICA_VS | QA_VS | 0.590942 | 0.026055 |
| TRP_Hipo | TRP_Plasma | 0.597261 | 0.024115 |
| KYN_Plasma | TRP_Plasma | 0.841933 | 0.000159 |
| KYN_Hipo | TRP_Plasma | 0.729975 | 0.003037 |
| GLU_Plasma | TRP_Plasma | 0.561707 | 0.036586 |
| GLU_VS | TRP_Fro | -0.55212 | 0.040641 |
| KYN_Hipo | KYN_Plasma | 0.699695 | 0.005342 |
| PICA_VS | KYN_Plasma | 0.600443 | 0.02318 |
| GLU_Plasma | KYN_Plasma | 0.540479 | 0.045998 |
| KYN_Fro | KYN_Hipo | 0.708435 | 0.00457 |
| GLU_Hypo | KYN_Hypo | 0.552705 | 0.040385 |
| PICA_Hipo | KYN_VS | -0.67797 | 0.007705 |
| GLU_VS | PICA_Plasma | 0.62387 | 0.017111 |
| PICA_VS | PICA_Hypo | -0.59397 | 0.025112 |
| GLU_VS | PICA_Fro | -0.54758 | 0.042674 |

**Table S5. Significant correlations among kynurenine pathway metabolites, tryptophan, and glutamate in female *Negr1^-/-^* mice.** Correlation data are shown for five regions: frontal cortex (Fro), hippocampus (Hipo), hypothalamus (Hypo), ventral striatum (VS), and blood plasma (Plasma). Seven metabolites most relevant to the study were included in the analysis: KYNA – kynurenic acid, KYN – kynurenine, TRP – tryptophan, QA – quinolinic acid, PICA – picolinic acid, XA – xanthurenic acid, and GLU – glutamate. Correlation = r-value.

| **Variable_1** | **Variable_2** | **Correlation** | **P_Value** |
| --- | --- | --- | --- |
| QA_Hipo | XA_Hipo | 0.579929 | 0.009174 |
| KYN_Hypo | XA_Hipo | -0.49451 | 0.02013 |
| QA_Hypo | XA_Hypo | 0.756827 | 0.005696 |
| GLU_Hypo | XA_Hypo | 0.77497 | 0.024292 |
| QA_Fro | XA_Fro | 0.856982 | 0.000512 |
| TRP_Fro | XA_Fro | 0.63519 | 0.031335 |
| KYN_Hypo | XA_Fro | 0.626909 | 0.010607 |
| GLU_Fro | XA_Fro | 0.806961 | 0.000749 |
| QA_VS | XA_VS | 0.672041 | 0.001717 |
| PICA_Plasma | XA_VS | 0.604395 | 0.037378 |
| GLU_VS | XA_VS | 0.910404 | 1.36E-05 |
| KYN_Fro | KYNA_Fro | 0.501611 | 0.034509 |
| PICA_VS | KYNA_VS | 0.693428 | 0.005634 |
| GLU_VS | KYNA_VS | 0.732663 | 0.003201 |
| TRP_Fro | QA_Plasma | -0.65507 | 0.013999 |
| GLU_Fro | QA_Plasma | -0.56387 | 0.036334 |
| TRP_Fro | QA_Hipo | -0.66883 | 0.049417 |
| KYN_Hypo | QA_Hipo | -0.56593 | 0.020493 |
| GLU_Hipo | QA_Hipo | 0.795657 | 0.003979 |
| **Variable_1** | **Variable_2** | **Correlation** | **P_Value** |
| TRP_Hipo | QA_Hypo | 0.687532 | 0.020054 |
| GLU_Hypo | QA_Hypo | 0.766569 | 0.001972 |
| QA_VS | QA_Fro | -0.62561 | 0.027548 |
| TRP_Fro | QA_Fro | 0.57507 | 0.027681 |
| KYN_Plasma | QA_Fro | 0.532915 | 0.033257 |
| KYN_Hypo | QA_Fro | 0.820008 | 0.001337 |
| PICA_Hypo | QA_Fro | 0.53558 | 0.036978 |
| GLU_Fro | QA_Fro | 0.733996 | 0.004563 |
| KYN_VS | QA_VS | 0.449625 | 0.024711 |
| PICA_Plasma | QA_VS | 0.690854 | 0.012858 |
| PICA_Hypo | QA_VS | -0.7071 | 0.013851 |
| GLU_Fro | QA_VS | -0.72552 | 0.024138 |
| GLU_VS | QA_VS | 0.67066 | 0.005671 |
| TRP_Hypo | TRP_Plasma | 0.583556 | 0.014665 |
| TRP_VS | TRP_Plasma | 0.410861 | 0.04227 |
| GLU_Plasma | TRP_Plasma | 0.50403 | 0.037605 |
| GLU_Hipo | TRP_Plasma | -0.66892 | 0.019567 |
| TRP_VS | TRP_Hypo | 0.609352 | 0.020493 |
| GLU_Hipo | TRP_Hypo | -0.81569 | 0.001116 |
| GLU_Hipo | TRP_Fro | -0.71606 | 0.005788 |
| GLU_Fro | TRP_Fro | 0.653524 | 0.013788 |
| KYN_Hypo | KYN_Plasma | 0.700072 | 0.006241 |
| GLU_Fro | KYN_Hypo | 0.581728 | 0.047674 |
| GLU_Fro | PICA_Plasma | -0.64214 | 0.024359 |
| GLU_VS | PICA_VS | 0.755694 | 0.002685 |


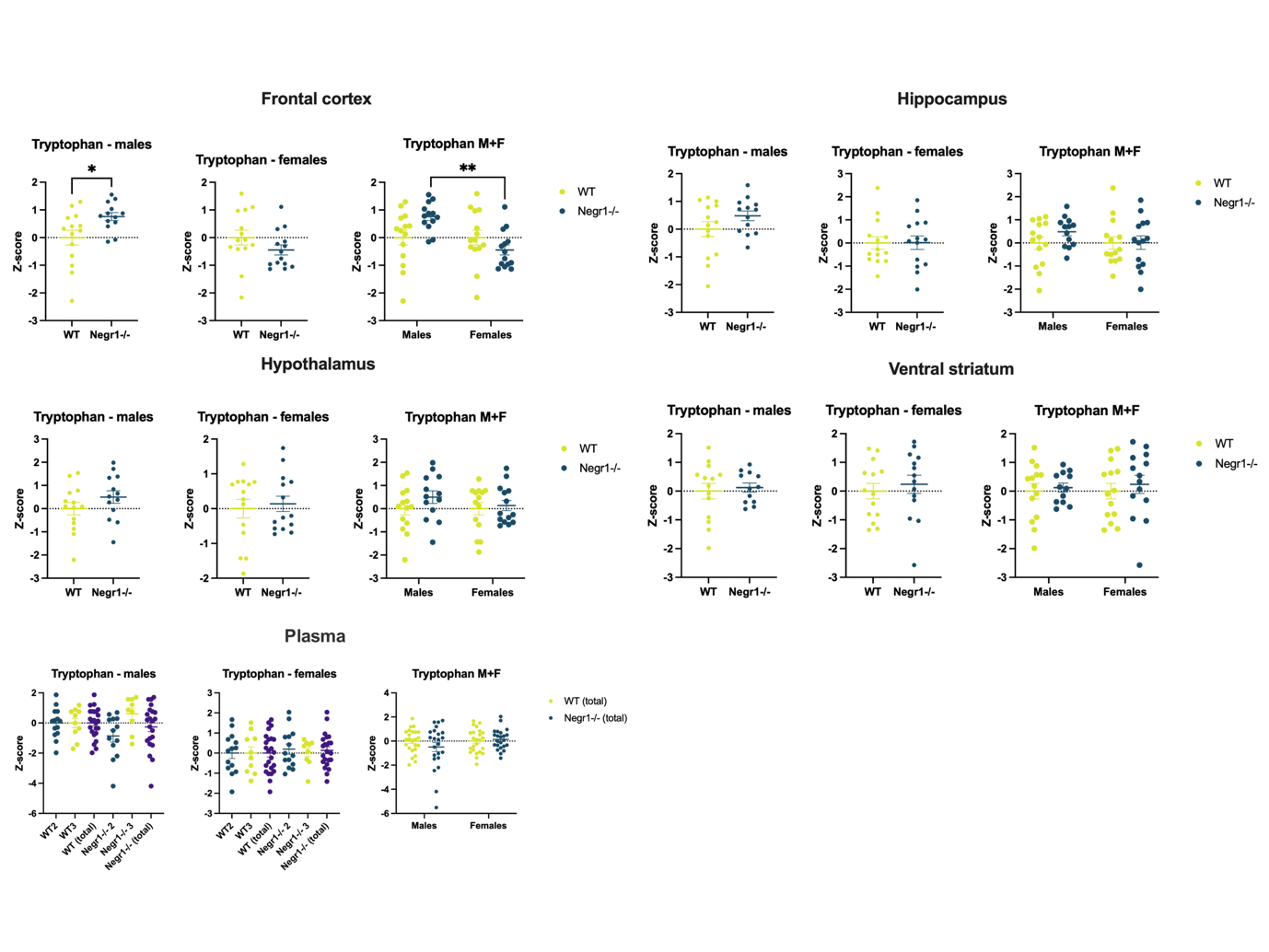


**Fig. S8. Differences in the tryptophan levels between wild-type (WT) and *Negr1*-deficient mice across four brain regions (frontal cortex, hippocampus, hypothalamus, ventral striatum) and blood plasma.** Significant differences in tryptophan levels were seen in the frontal cortex between male WT and *Negr1^-/-^* mice, and between male and female *Negr1^-/-^* mice. Notably, tryptophan levels were elevated in *Negr1^-/-^* males while showing a slight decrease in *Negr1^-/-^* females. Data are presented as mean ± SEM, n = 12 - 14. Plasma data were obtained from cohort 2 (5-month-olds) and cohort 3 (7-month-olds); brain data are from cohort 2 only. Statistical analysis: unpaired *t*-test for separate male and female genotype comparisons, one-way ANOVA for plasma, and ordinary two-way ANOVA with Tukey’s HSD test for combined-sex analysis. * – *p* < 0.05, ** – *p* < 0.001.


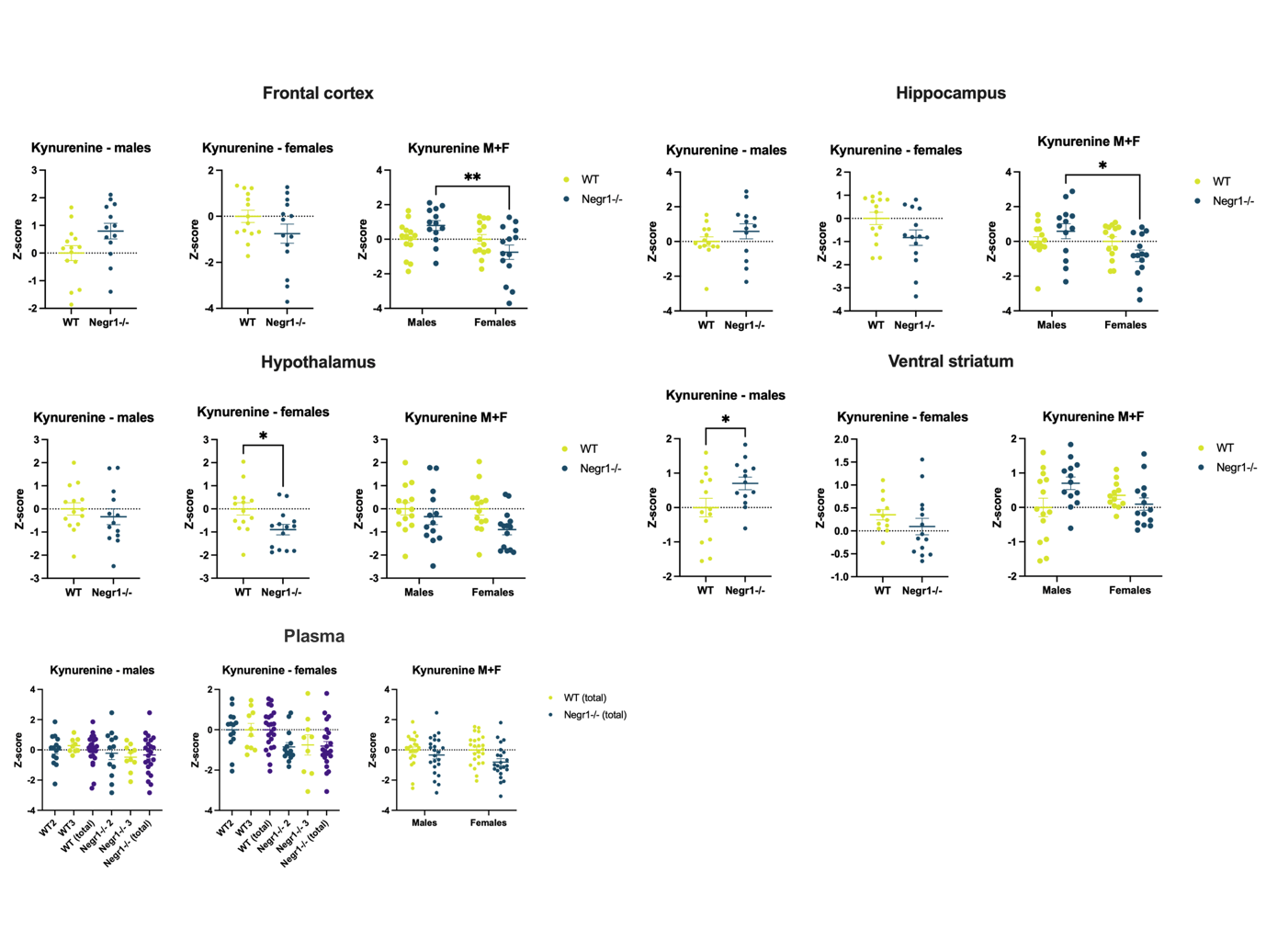


**Fig. S9. Differences in the kynurenine levels between wild-type (WT) and *Negr1*-deficient mice across four brain regions (frontal cortex, hippocampus, hypothalamus, ventral striatum) and blood plasma.** Most notable differences were between male and female *Negr1*-deficient mice in the frontal cortex and hippocampus, where kynurenine levels were increased in males and decreased in females. Although the difference was not statistically significant compared to WT mice, it was significant between genders. In the hypothalamus, there was a significant decrease in the kynurenine levels of female *Negr1^-/-^* mice and in the ventral striatum, there was a significant increase in male *Negr1^-/-^* mice. Data represents mean ± SEM, n = 12 - 14. Plasma data is from cohort 2 (5-month-olds) and cohort 3 (7-month-olds); brain data is only from cohort 2. Unpaired t-test was used for the separate male and female genotype analysis (one-way ANOVA for plasma) and ordinary two-way ANOVA (Tukey HSD test) when both sexes were together. * - p-value < 0.05, ** - p-value < 0.001.


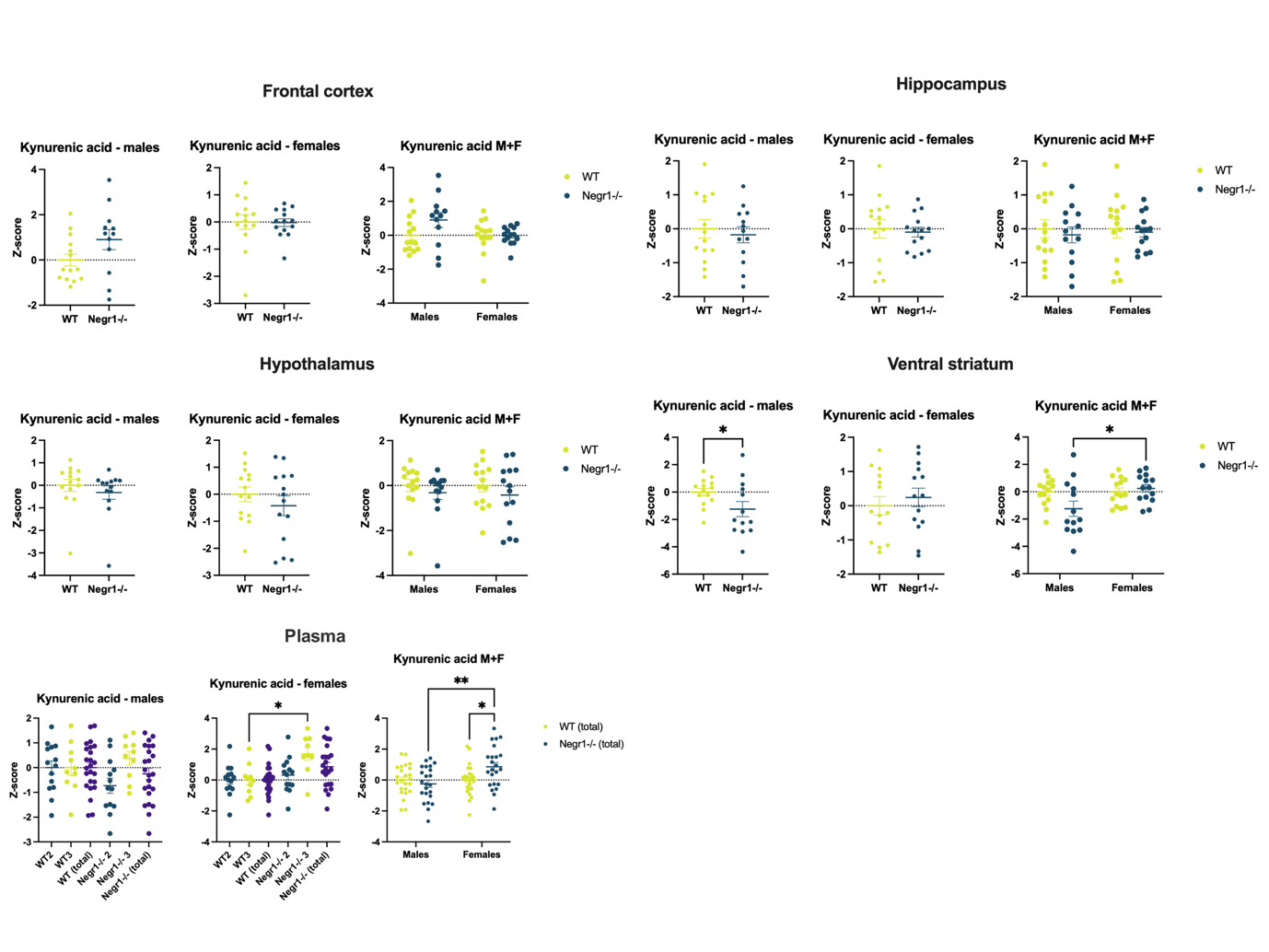


**Fig. S10. Differences in the kynurenic acid levels between wild-type (WT) and *Negr1*-deficient mice across four brain regions (frontal cortex, hippocampus, hypothalamus, ventral striatum) and blood plasma.** In the ventral striatum, the kynurenic acid levels were significantly decreased in male *Negr1^-/-^* mice, while no difference was observed between WT and mutant female mice. In contrast, plasma kynurenic acid levels were significantly increased in female *Negr1^-/-^* mice, with no corresponding difference between WT and *Negr1^-/-^* males. Data represents mean ± SEM, n = 12 - 14. Plasma data is from cohort 2 (5-month-olds) and cohort 3 (7-month-olds); brain data is only from cohort 2. Unpaired t-test was used for the separate male and female genotype analysis (one-way ANOVA for plasma) and ordinary two-way ANOVA (Tukey HSD test) when both sexes were together. * - p-value < 0.05, ** - p-value < 0.001.


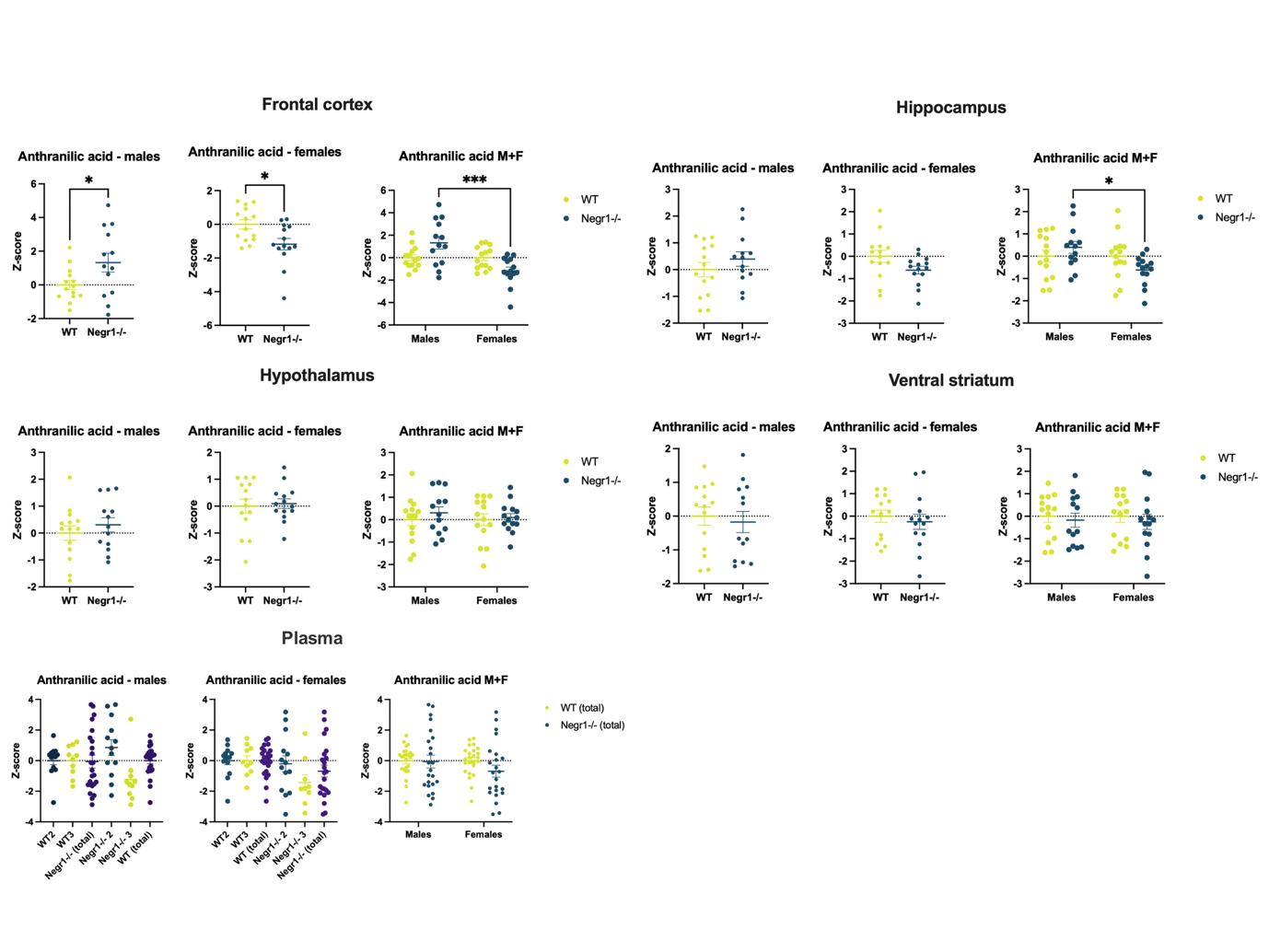


**Fig. S11. Differences in the anthranilic acid levels between wild-type (WT) and *Negr1*-deficient mice across four brain regions (frontal cortex, hippocampus, hypothalamus, ventral striatum) and blood plasma.** In the frontal cortex, the anthranilic acid levels had significantly increased in male and decreased in female *Negr1*-deficient mice. The same trend could be seen in the hippocampus. Data represents mean ± SEM, n = 12 - 14. Plasma data is from cohort 2 (5-month-olds) and cohort 3 (7-month-olds); brain data is only from cohort 2. Unpaired t-test was used for the separate male and female genotype analysis (one-way ANOVA for plasma) and ordinary two-way ANOVA (Tukey HSD test) when both sexes were together. * - p-value < 0.05, *** - p-value < 0.0001.


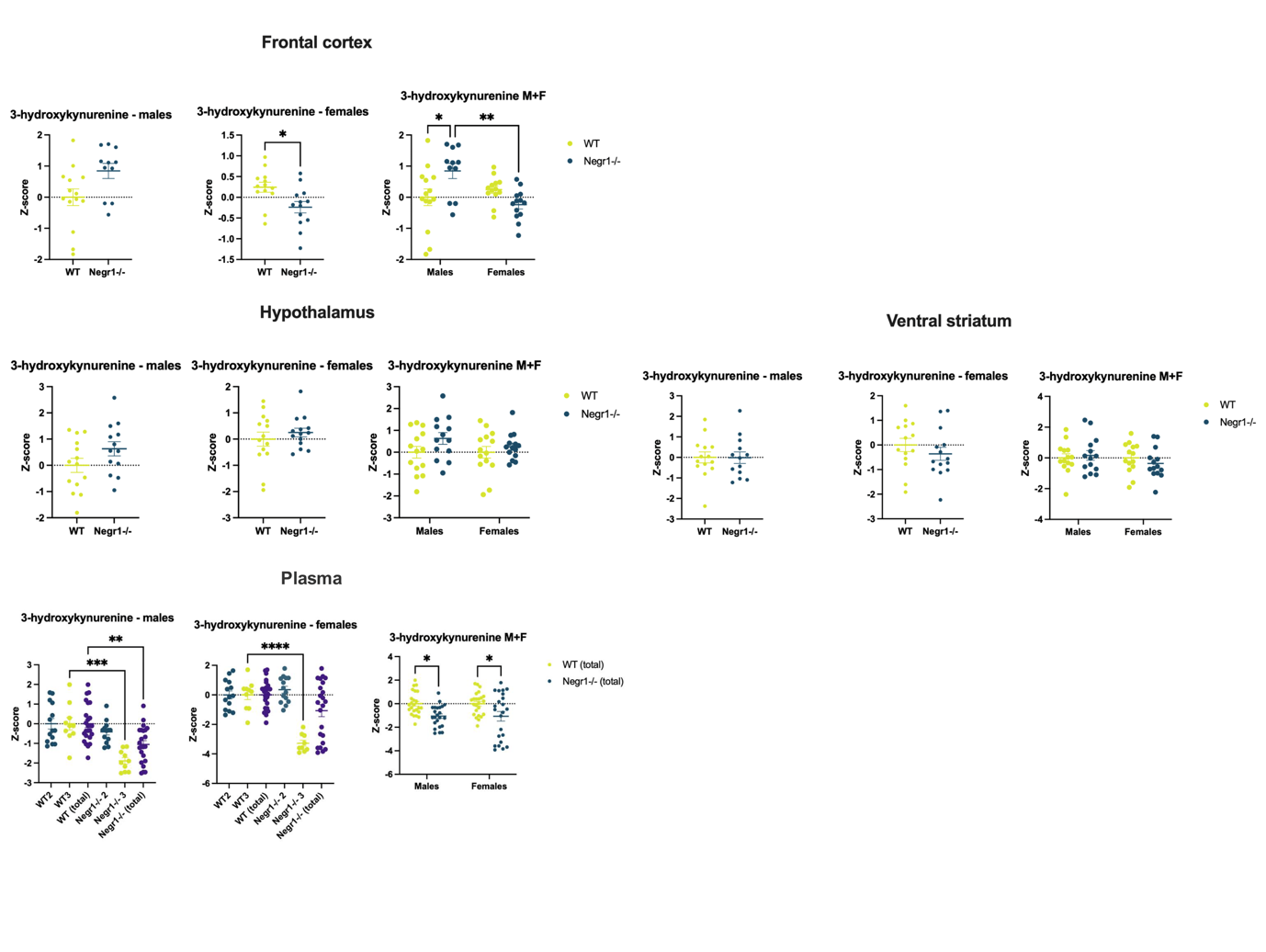


**Fig. S12. Differences in the 3-hydroxykynurenine levels between wild-type (WT) and *Negr1*-deficient mice across three brain regions (frontal cortex, hypothalamus, ventral striatum) and blood plasma.** 3-hydroxykynurenine displayed interesting results in the frontal cortex - metabolite levels were increased in male *Negr1^-/-^* mice but decreased in female *Negr1^-/-^* mice compared to the WT mice. In the blood plasma, 3-hydroxykynurenine levels were significantly diminished in both male and female *Negr1^-/-^* mice from cohort 3. In the hippocampus, the results were not up to standard and were excluded. Data represents mean ± SEM, n = 12 - 14. Plasma data is from cohort 2 (5-month-olds) and cohort 3 (7-month-olds); brain data is only from cohort 2. Unpaired t-test was used for the separate male and female genotype analysis (one-way ANOVA for plasma) and ordinary two-way ANOVA (Tukey HSD test) when both sexes were together. * - p-value < 0.05, ** - p-value < 0.001, *** - p-value < 0.0001, **** - p-value < 0.00001.


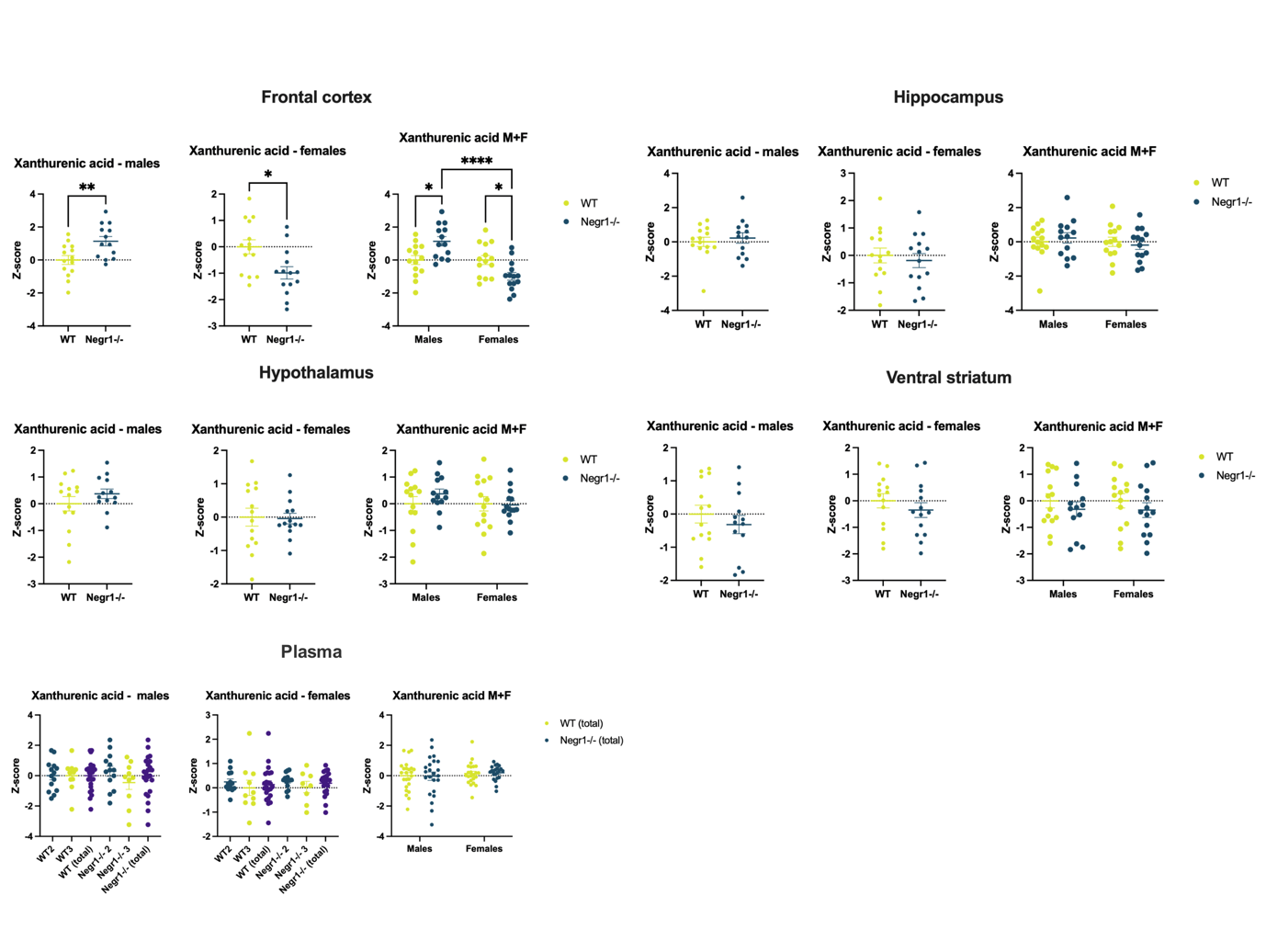


**Fig. S13. Differences in the xanthurenic acid levels between wild-type (WT) and *Negr1*-deficient mice across four brain regions (frontal cortex, hippocampus, hypothalamus, ventral striatum) and blood plasma.** Like with most other kynurenine pathway metabolites, xanthurenic acid levels were increased in male *Negr1^-/-^* mice, while the opposite was true for female mice in the frontal cortex. Data represents mean ± SEM, n = 12 - 14. Plasma data is from cohort 2 (5-month-olds) and cohort 3 (7-month-olds); brain data is only from cohort 2. Unpaired t-test was used for the separate male and female genotype analysis (one-way ANOVA for plasma) and ordinary two-way ANOVA (Tukey HSD test) when both sexes were together. * - p-value < 0.05, ** - p-value < 0.001. **** - p-value < 0.00001.


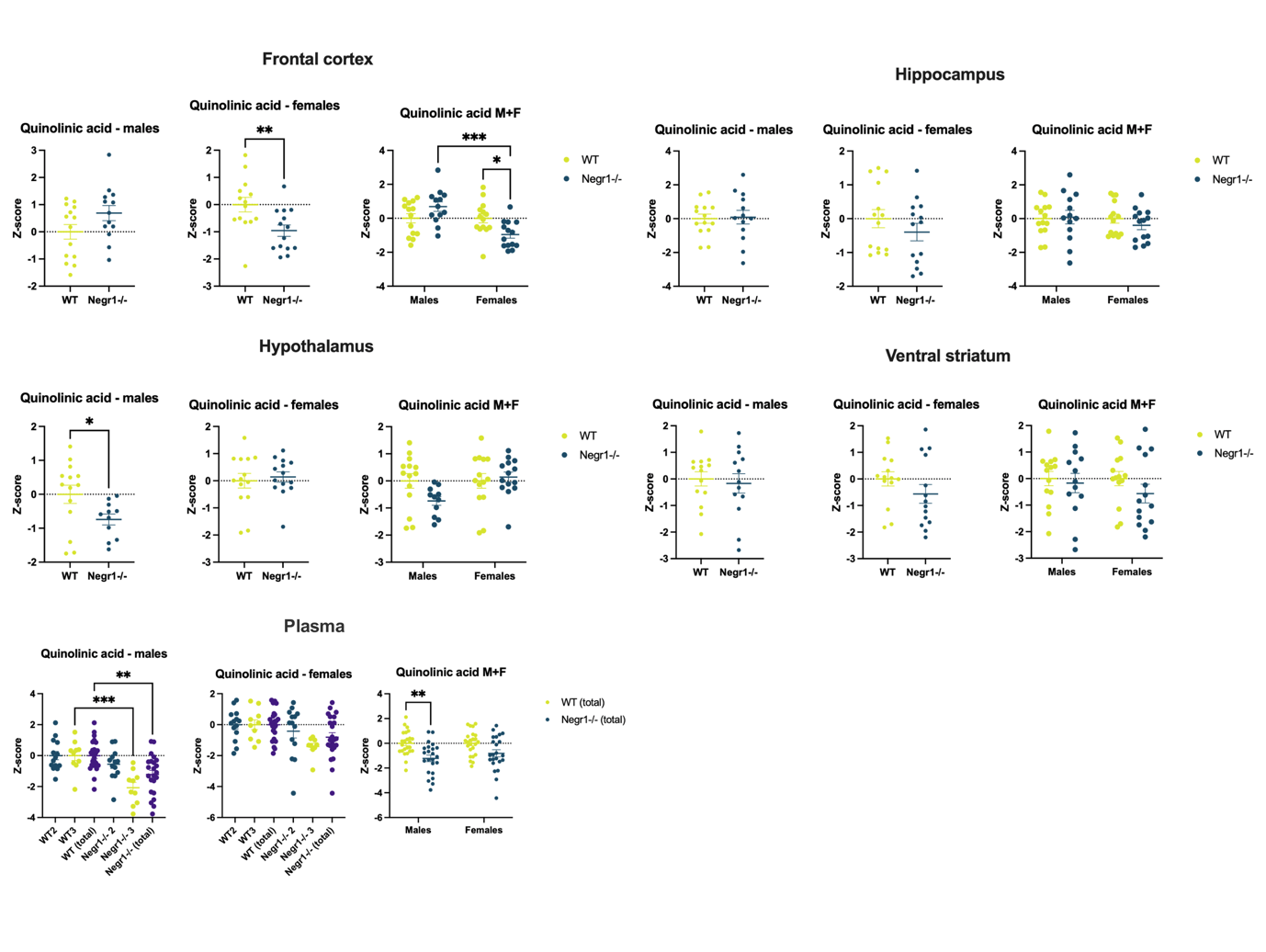


**Fig. S14. Differences in the quinolinic acid levels between wild-type (WT) and *Negr1*-deficient mice across four brain regions (frontal cortex, hippocampus, hypothalamus, ventral striatum) and blood plasma.** In the frontal cortex, the quinolinic acid levels were significantly decreased in female *Negr1^-/-^* mice but slightly increased in males. However, in the hypothalamus and blood plasma, quinolinic acid levels were strongly decreased in male *Negr1^-/-^* mice, while there was no change in the female metabolite levels. Data represents mean ± SEM, n = 12 - 14. Plasma data is from cohort 2 (5-month-olds) and cohort 3 (7-month-olds); brain data is only from cohort 2. Unpaired t-test was used for the separate male and female genotype analysis (one-way ANOVA for plasma) and ordinary two-way ANOVA (Tukey HSD test) when both sexes were together. * - p-value < 0.05, ** - p-value < 0.001. *** - p-value < 0.0001.


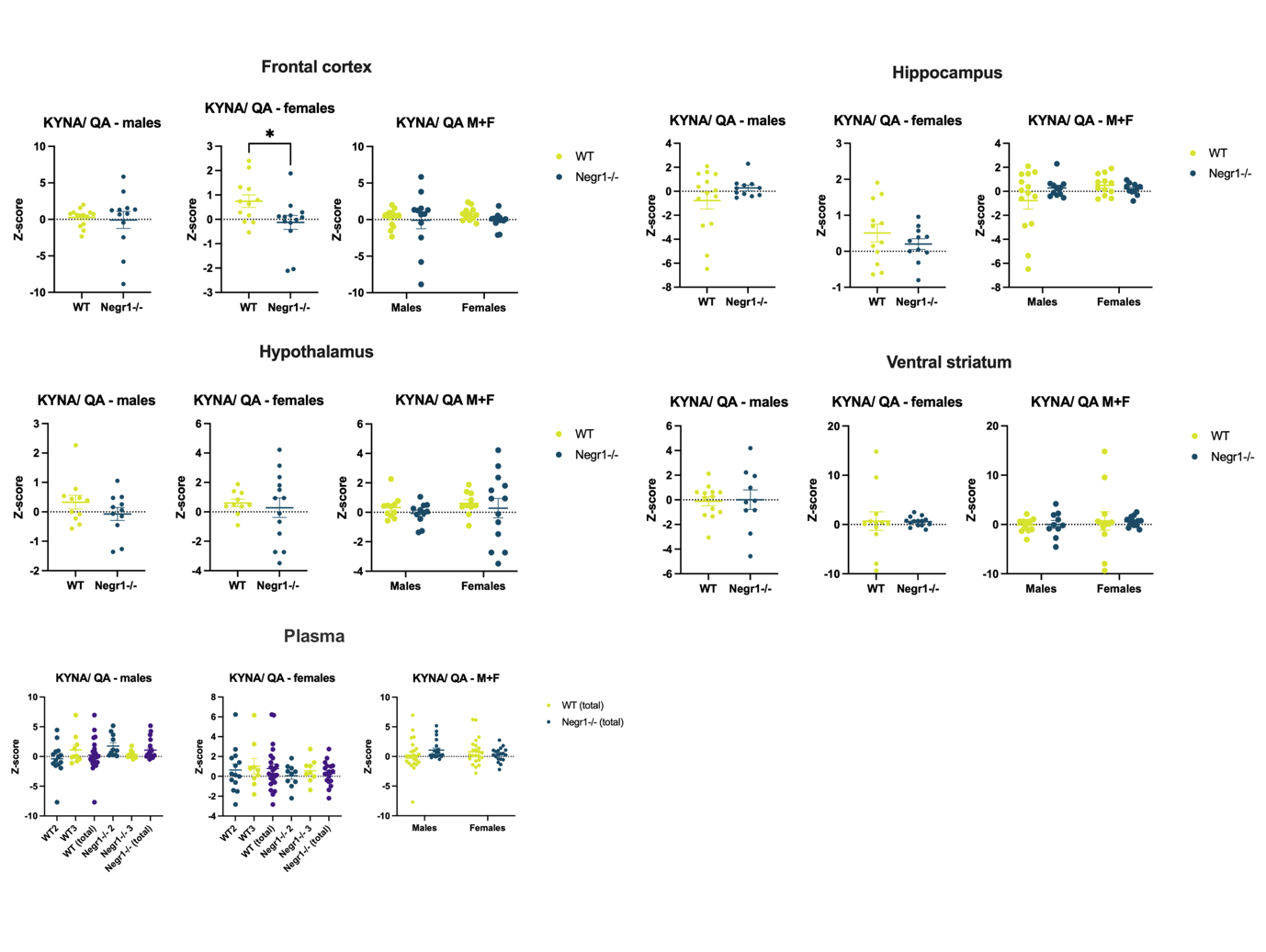


**Fig. S15. Differences in the kynurenic acid/ quinolinic acid ratio between wild-type (WT) and *Negr1*-deficient mice across four brain regions (frontal cortex, hippocampus, hypothalamus, ventral striatum) and blood plasma.** The only significant difference in the kynurenic acid/ quinolinic acid ratio was in the frontal cortex of female mice - there was a significant decrease in the ratio of *Negr1^-/-^* mice compared to WT mice. Data represents mean ± SEM, n = 12 - 14. Plasma data is from cohort 2 (5-month-olds) and cohort 3 (7-month-olds); brain data is only from cohort 2. Unpaired t-test was used for the separate male and female genotype analysis (one-way ANOVA for plasma) and ordinary two-way ANOVA (Tukey HSD test) when both sexes were together. * - p-value < 0.05.


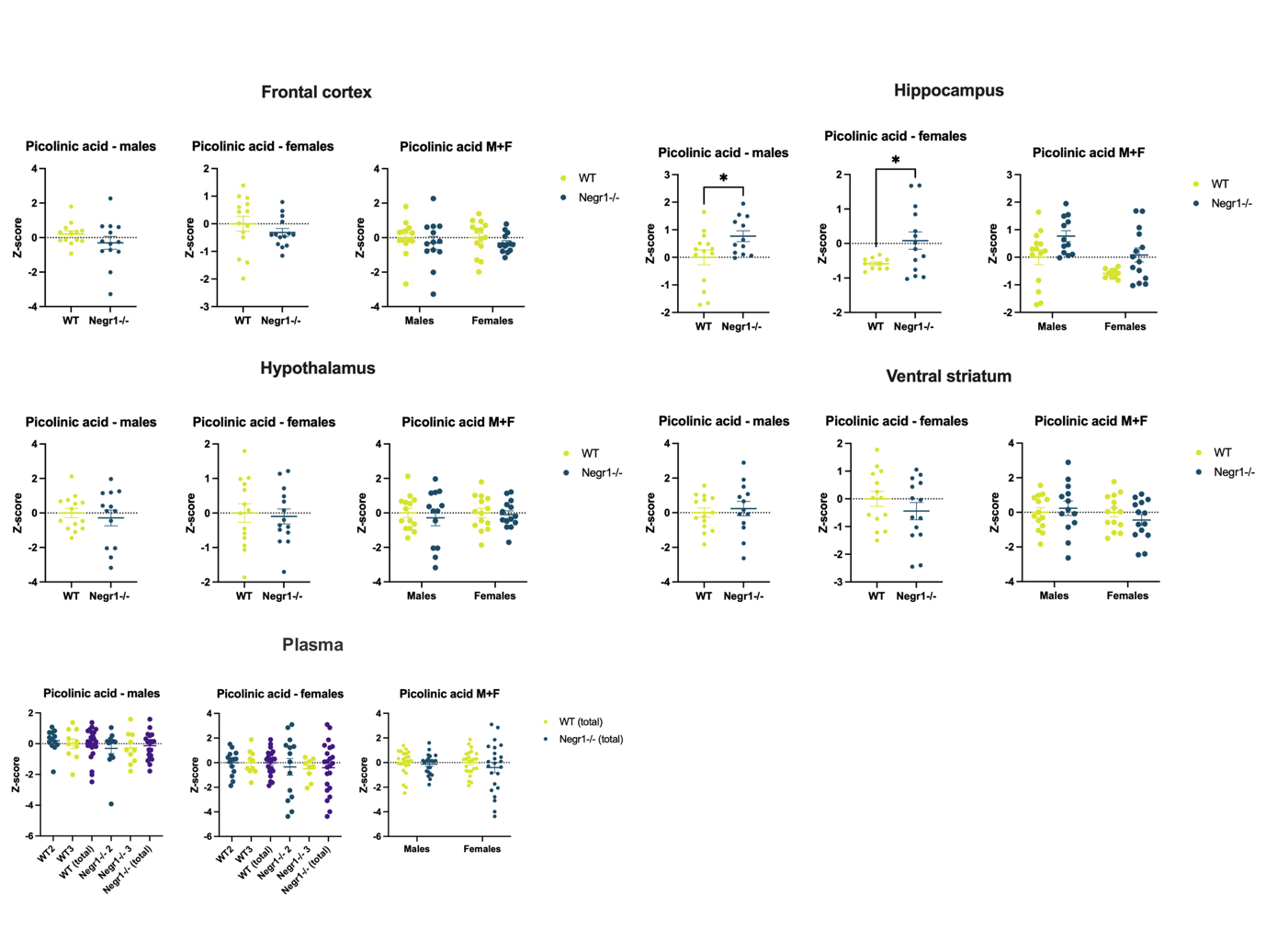


**Fig. S16. Differences in the picolinic acid levels between wild-type (WT) and *Negr1*-deficient mice across four brain regions (frontal cortex, hippocampus, hypothalamus, ventral striatum) and blood plasma.** Picolinic acid levels were increased in both male and female *Negr1^-/-^* mice in the hippocampus compared to their WT counterparts. Data represents mean ± SEM, n = 12 - 14. Plasma data is from cohort 2 (5-month-olds) and cohort 3 (7-month-olds); brain data is only from cohort 2. Unpaired t-test was used for the separate male and female genotype analysis (one-way ANOVA for plasma) and ordinary two-way ANOVA (Tukey HSD test) when both sexes were together. * - p-value < 0.05.


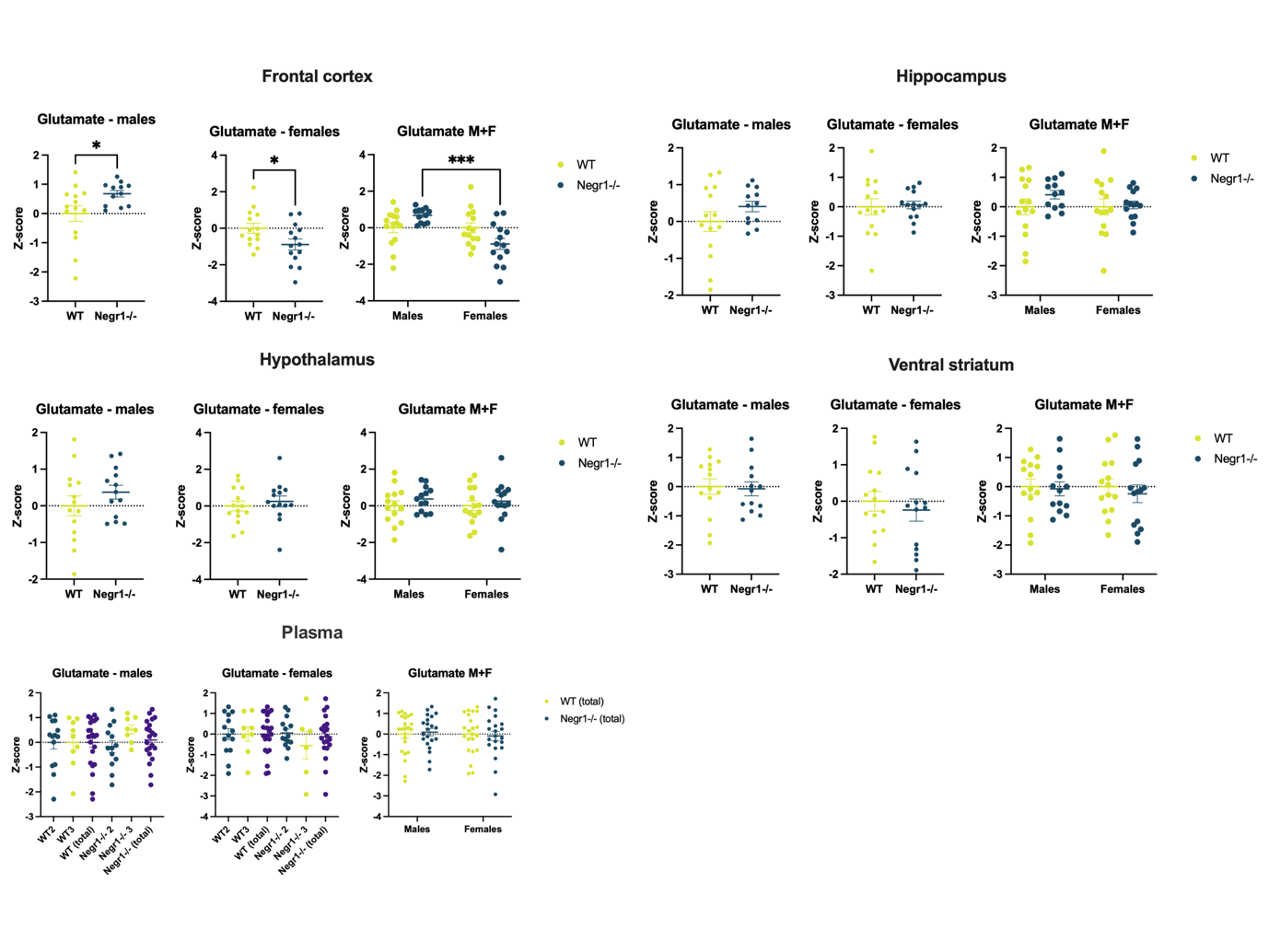


**Fig. S17. Differences in the glutamate levels between wild-type (WT) and *Negr1*-deficient mice across four brain regions (frontal cortex, hippocampus, hypothalamus, ventral striatum) and blood plasma.** Similarly to most kynurenine pathway metabolites, glutamate levels were significantly increased in male *Negr1^-/-^* mice and decreased in females in the frontal cortex. Data represents mean ± SEM, n = 12 - 14. Plasma data is from cohort 2 (5-month-olds) and cohort 3 (7-month-olds); brain data is only from cohort 2. Unpaired t-test was used for the separate male and female genotype analysis (one-way ANOVA for plasma) and ordinary two-way ANOVA (Tukey HSD test) when both sexes were together. * - p-value < 0.05, *** - p-value < 0.0001.

**Table S6. Primer sequences.**

| Hprt_mm_F | GCAGTACAGCCCCAAAATGG |
| --- | --- |
| Hprt_mm_R | AACAAAGTCTGGCCTGTATCCAA |
| Grin1_mm_F | GTTCTTCCGCTCCGGCTTTG |
| Grin1_mm_R | CGAACCCATGTCTTATCCAGGTC |
| Grin2a_mm_F | TGCAAGTTACACAGCCAACCTG |
| Grin2a_mm_R | ATCGGAAAGGCGGAGAATAGTC |
| Grin2b_mm_F | TCATTTCTGCTCAGACTCTCACC |
| Grin2b_mm_R | TCAATGGATGGGCCAAACTGG |
| Srr_mm_F | AGAAGCCCAAAGCCGTAGTTAC |
| Srr_mm_R | TGCAGTTGGGAGCTGTTTGG |

**ARRIVE guidelines checklist**

1. **Study design. For each experiment, provide brief details of the study design, including:**
   1. **The groups being compared, including control groups. If no control group has been used, the rationale should be stated.**

**Ans:** The *Negr1*^-/-^ mice were compared with their littermate wild-type (WT) controls. Both genotypes received either chronic MK-801 or saline injections. Thus, the experimental groups included WT-saline, WT–MK-801, KO-saline, and KO–MK-801 mice. Behavioural activity was assessed daily in the open field following each injection to monitor the development of behavioural sensitisation.

- 1. **The experimental unit (e.g. a single animal, litter, or cage of animals).**

**Ans:** *Negr1*^-/-^ and WT mice used in this study were littermates. Each group consisted of 10 animals per cage.

1. **Sample size**
   1. **Specify the exact number of experimental units allocated to each group, and the total number in each experiment. Also, indicate the total number of animals used**.

**Ans:**

**Cohort 1:** A total of 92 mice were used in the MK-801 experiment: 46 *Negr1*^-/-^ animals (20 males, 26 females) and 46 wild-type (WT) controls (20 males, 26 females). Of these, 52 mice received MK-801 (10 *Negr1*^-/-^ males, 16 *Negr1*^-/-^ females, 10 WT males, 16 WT females), and 40 mice received physiological saline (10 *Negr1*^-/-^ males, *Negr1*^-/-^ females, 10 WT males, 10 WT females).

The increased number of female mice in the MK-801-treated groups was based on prior observations in males, which showed a zig-zag locomotor response pattern following MK-801 administration, suggesting a higher variability in treatment response. Because saline-treated mice displayed consistent behavioural outcomes, and the open-field system had a capacity limit of 48 mice per experimental run, the number of saline-treated females was reduced to 8 per group, while the number of MK-801-treated females was increased to 16 per group. This allocation strategy prioritised adequate statistical power to detect behavioural effects of MK-801 and potential genotype-dependent differences.

For subsequent gene expression analyses, a minimum of 10 mice per group was required based on prior results from our lab. Therefore, four additional female mice (2 WT and 2 *Negr1*^-/-^) received saline and were placed in an identical open-field apparatus without behavioural recording for 30 minutes. As a result, gene expression groups consisted of 10 animals per genotype and treatment group.

**Cohort 2:** A total of 56 mice were used in the experiment. 28 *Negr1*^-/-^ animals (14 male + 14 female mice) and 28 control WT animals (14 male + 14 female mice).

**Cohort 3:** A total of 39 mice were used in the experiment. 20 *Negr1*^-/-^ animals (10 male + 9 female mice) and 20 control WT animals (10 male + 10 female mice).

- 1. **Explain how the sample size was decided. Provide details of any a priori sample size calculation, if done.**

**Ans:** Group sizes for both sexes, were determined based on prior studies employing similar paradigms and in accordance with the 3Rs principle (Replacement, Reduction, and Refinement) to minimise animal use. Therefore, the group size was set based on the expected variability of the data as suggested in the National Centre for the Replacement, Refinement and Reduction of Animals in Research (NC3Rs). (2019). The 3Rs (<https://www.nc3rs.org.uk/the-3rs>).

1. **Inclusion and exclusion criteria**
   1. **Describe any criteria used for including and excluding animals (or experimental units) during the experiment, and data points during the analysis. Specify if these criteria were established a priori. If no criteria were set, state this explicitly**.

**Ans:** Animals were excluded if they developed serious health issues, such as inflammation on the skin or tail, rapid weight loss (≥ 20%; monitored at least weekly or daily if needed), or signs of dehydration. No animals met these criteria in the current study, and no exclusions were made.

During data analysis, outliers were identified and excluded using the ROUT method in GraphPad Prism (qPCR, metabolites) or R (behaviour).

- 1. **For each experimental group, report any animals, experimental units or data points not included in the analysis and explain why. If there were no exclusions, state so.**

**Ans:** In cohort 3, kynurenine pathway data from one female mouse from the *Negr1*^-/-^ group was excluded due to abnormally low values resulting from a technical error. No animals were excluded for health-related reasons. During statistical analysis, typically 1-2 (maximum of three) mice per group were excluded based on the results of the outlier test.

- 1. **For each analysis, report the exact value of n in each experimental group.**

**Ans:**

**Behaviour:**46 *Negr1*^-/-^ mice (20 males, 26 females) and 46 WT mice (20 males, 26 females).
52 mice received MK-801 (10 *Negr1*^-/-^ males, 16 *Negr1*^-/-^ females, 10 WT males, 16 WT females) and 40 received saline (10 *Negr1*^-/-^ males, 10 *Negr1*^-/-^ females, 10 WT males, 10 WT females). The behaviour of four saline-treated female mice (2 *Negr1*^-/-,^ 2 WT) was not recorded due to throughput limitations of the behavioural apparatus.

**qPCR:**

A total of 40 *Negr1*^-/-^ mice (20 males, 20 females) and 40 WT mice (20 males, 20 females) were included.

Among them, 40 mice received MK-801 (10 *Negr1*^-/-^ males, 10 *Negr1*^-/-^ females, 10 WT males, 10 WT females) and 40 received saline (10 *Negr1*^-/-^ males, 10 *Negr1*^-/-^ females, 10 WT males, 10 WT females).

All males included in the behavioural study were also used for qPCR analysis. For females, 10 animals from each of the 16 MK-801–treated genotypes were randomly selected for qPCR analysis.

**Brain metabolites analysis:** 28 *Negr1*^-/-^ animals (14 male + 14 female mice) and 28 control WT animals (14 male + 14 female mice).

**Blood metabolites analysis:** 56 animals from cohort 2. 28 *Negr1*^-/-^ animals (14 male + 14 female mice) and 28 control WT animals (14 male + 14 female mice). 39 animals from cohort 3. 20 *Negr1*^-/-^ animals (10 male + 9 female mice) and 20 control WT animals (10 male + 10 female mice).

1. **Randomisation**
   1. **State whether randomisation was used to allocate experimental units to control and treatment groups. If done, provide the method used to generate the randomisation sequence.**

**Ans:** All experimental animals were assigned to four groups by matching body weights within the treatment groups.

To minimise potential allocation and experimental bias, several measures were implemented. All animals were maintained under identical housing conditions, with treatment assignments balanced across cages to prevent location-based effects. Behavioural testing and sample collection were conducted in a randomised order (Control WT, Control *Negr1^-/-^*, MK-801 WT, MK-801 *Negr1^-/-^*) according to the assigned animal codes to minimise systematic bias.

- 1. **Describe the strategy used to minimise potential confounders, such as the order of treatments and measurements, or animal/cage location. If confounders were not controlled, state this explicitly.**

**Ans:** Randomisation of treatment order is explained in section 4a.

The order of behavioural tests, qPCR, and mass-spectrometry measurements was randomised across the groups to prevent systematic order effects.

**Balanced Cage Location**

**Ans:** Animals were housed in standard conditions with cages distributed evenly and in a randomised manner based on the assigned codes, across racks to avoid location-based environmental differences (e.g., light, temperature).

**Consistent Timing**

**Ans:** All measurements were performed at similar times of day to control for circadian influences.

**Equal Handling**

**Ans:** Animals were handled similarly before tests to reduce stress-related variability.

1. **Blinding**

**Describe who was aware of the group allocation at the different stages of the experiment (during the allocation, the conduct of the experiment, the outcome assessment, and the data analysis).**

**Ans:** During allocation and experimental conduct, two individuals were aware of group assignments: one responsible for cage-level identification and the other for administering MK-801 or saline. All other experimenters conducting behavioural testing were blinded to genotype and treatment. Behavioural outcomes were recorded automatically by the apparatus without human intervention. Data analysis was performed in parallel by multiple researchers (CK, EV, MAP), who were blinded to group allocation to ensure consistency and minimise bias.

1. **Outcome measures**
2. **Clearly define all outcome measures assessed (e.g. cell death, molecular markers, or behavioural changes).**

**Open field test:** total distance covered, distance covered in corners, rotations (clockwise and counterclockwise), total time spent moving, corner visits, and number of rearings.

**Molecular markers:**

Investigated through RT-qPCR in frontal cortex, hippocampus, ventral striatum, and hypothalamus: *Grin1, Grin2a, Grin2b, Srr, (HPRT as housekeeper).*

Investigated through high-performance liquid chromatography-mass spectrometry in frontal cortex, hippocampus, ventral striatum and hypothalamus: tryptophan, kynurenine, kynurenic acid, xanthurenic acid, quinolinic acid, anthralinic acid, picolinic acid, 3-hydroxykynurenine, glutamate.

1. **For hypothesis-testing studies, specify the primary outcome measure, i.e. the outcome measure that was used to determine the sample size.**

**Ans:** The primary behavioural outcome measure of interest was total distance travelled (in meters). However, this study was exploratory in nature; therefore, no formal sample size calculation was performed, and no single predefined primary outcome was used to determine group size. Group sizes were determined based on previous results from the lab on this mouseline and on 3R principles. Because behavioural variability was higher in male MK-801–treated mice, compared to the saline-treated group, the number of treated females was increased based on prior experience with variance. Under standard conditions, we use groups of approximately 10 mice, which have proven sufficient to detect genotype-dependent effects in gene-deficient mouse models.

**7. Statistical methods**

1. **Provide details of the statistical methods used for each analysis, including software used.**

**Ans:**

Data are presented as mean values ± standard error of the mean (SEM). Before the analyses, an outlier test, using the ROUT method, was performed on all the data. Log-transformation was used to normalise the data before analysis. Normality of data distribution was assessed using the Shapiro–Wilk test. Brain metabolite levels were analysed using Student’s *t*-test or the Mann–Whitney *U* test for non-parametric data. Blood plasma metabolites and qPCR data were evaluated using two-way ANOVA followed by Tukey’s post hoc test. For the supplementary data, one-way ANOVA was used for blood plasma to allow pooling of the data.

Statistical analyses for behavioural experiments and metabolite measurements, as well as correlation plot generation, were conducted using R (version 4.3.1). Analysis of qPCR data and generation of all other graphs (excluding correlation plots) were performed using GraphPad Prism (version 10.2.1). Z-scores were calculated for each sample when necessary to standardise and compare data across groups (between different brain regions and blood serum) using the mean and standard deviation of the control group,

$z=\frac{x_{i}-\mu_{control}}{\sigma_{control}}$, where x_i_ is the log₂-transformed value for each subject, 𝜇 the group mean, and 𝜎 the standard deviation. Statistical significance was defined as *p* < 0.05.

1. **Describe any methods used to assess whether the data met the assumptions of the statistical approach, and what was done if the assumptions were not met.**

**Ans:** For the statistical approach, the data were tested for normality and lognormality using the Shapiro–Wilk test. When the data met the normality and homogeneity of variance, parametric tests such as Student’s *t*-test or two-way ANOVA followed by a Tukey’s post hoc test were performed.

When these assumptions were not met, non-parametric tests were used instead. Statistical analyses for behavioural experiments and metabolite measurements were conducted using R (version 4.3.1). Analysis of qPCR data was performed using GraphPad Prism (version 10.2.1).

**8. Experimental animals**

1. **Provide species-appropriate details of the animals used, including species, strain and substrain, sex, age or developmental stage, and, if relevant, weight.**

**Ans:**

**Species:** *Mus musculus*

**Strain: Animal** background (129S5/SvEvBrd × C57BL/6N) × (129S5/SvEvBrd × C57BL/6N)

**Sex:** Males and females

**Age:** Cohort 1: 2 months

Cohort 2: 5 months

Cohort 3: 7 months

1. **Provide further relevant information on the provenance of animals, health/immune status, genetic modification status, genotype, and any previous procedures**

**Ans:** Animals used in this manuscript were male and female wild-type (WT) and their *Negr1*-deficient littermates (*Negr1*^-/-^) in F2 background [(129S5/SvEvBrd × C57BL/6) × (129S5/SvEvBrd × C57BL/6)], as described in Lee et al. (2012). These transgenic mice were obtained from Michael K. E. Schäfer, Department of Anesthesiology, University Medical Center of Johannes Gutenberg-University, 55131 Mainz, Germany.

The general health and immune status of the mice were normal, and the genetic modification did not produce any adverse effects on their overall health. Genotyping was performed as described by Lee et al. (2012).

No prior experimental procedures were performed on the cohorts used in this manuscript.

**9. Experimental procedures**

**For each experimental group, including controls, describe the procedures in enough detail to allow others to replicate them, including:**

1. **What was done, how it was done and what was used.**

**Ans:** Mice were transferred from the breeding facility to the holding room at 7–8 weeks of age. Body weights were recorded every two weeks, with measurements paused one week prior to the start of the experiments. During this period, the general health and body weight of the animals were monitored to facilitate appropriate assignment into treatment and control groups. Based on the genotype, the first cohort was divided into four groups: Control WT, Control *Negr1^-/-^*, MK-801 WT, MK-801 *Negr1^-/-^*. The second and third cohorts consisted of two groups: WT and *Negr1^-/-^*. All the mice were group-housed in cages with 10 mice per cage and were maintained under the same food, water, temperature, and light conditions. All other details are mentioned in the manuscript under the 'Methods' section.

1. **When and how often.**

**Ans:** An open-field test, which included MK-801 treatment, was conducted for 9 consecutive days with the male mice and 5 consecutive days with the female mice. All other details are mentioned in the manuscript under the 'Methods' section.

1. **Where (including details of any acclimatisation periods).**

**Ans:** Acclimatisation period: Animals were kept at least for 1 hour in the designated procedure room before the start of the behavioural experiment (open field test). All procedures were performed in the designated rooms of the animal facility for each procedure.

1. **Why (provide rationale for procedures).**

**Ans:** A 1-hour acclimatisation period was included before behavioural testing to reduce stress caused by environmental changes during the mice’s inactive (light) phase. The open field test was chosen to test the psychomotor activity, exploration behaviour, motivation, anxiety and cognitive processing of the mice, which are often seen impaired in psychiatry-disease models. MK-801 treatment was chosen because it’s a non-competitive NMDA receptor antagonist used to model glutamatergic imbalances observed in neuropsychiatric and neurodegenerative conditions. The rationale for these procedures is provided in more detail in the discussion section of the manuscript.

**10. Results**

**For each experiment conducted, including independent replications, report:**

1. **Summary/descriptive statistics for each experimental group, with a measure of variability where applicable (e.g. mean and SD, or median and range).**

**Ans:** For each experimental group, data are presented as mean ± SEM. The number of animals per group ranged from 8 to 10. Descriptive statistics (mean ± SEM) are presented for each experimental group in Figures 1–6 and Supplementary Figures S1–S8.

1. **If applicable, the effect size with a confidence interval.**

**Ans:** Not applicable.

**The Recommended Set**

**11. Abstract**

**Provide an accurate summary of the research objectives, animal species, strain and sex, key methods, principal findings, and study conclusions.**

Objective: first paragraph.

Strain, sex and key methods: second paragraph.

Principal findings: third paragraph.

Conclusion: third paragraph.

**12. Background**

1. **Include sufficient scientific background to understand the rationale and context for the study, and explain the experimental approach.**

Background: introduction in the manuscript.

Experimental approach: methods in the manuscript.

1. **Explain how the animal species and model used address the scientific objectives and, where appropriate, the relevance to human biology.**

Mice were chosen as the model organism due to their well-characterised genetics, short lifespan, and strong homology with human neurobiology. *Negr1* knockout mice provide a relevant model for studying molecular mechanisms underlying neuropsychiatric disorders.

Introduction in the manuscript, paragraphs 1-5.

**13. Objectives**

**Clearly describe the research question, research objectives and, where**

**appropriate, specific hypotheses being tested.**

Research question: introduction in the manuscript, paragraph 6.

Research Objective: introduction in the manuscript, paragraph 7.

Hypothesis test: introduction in the manuscript, paragraph 7.

**14. Ethical statement**

**Provide the name of the ethical review committee or equivalent that has approved the use of animals in this study, and any relevant licence or protocol numbers (if applicable). If ethical approval was not sought or granted, provide a justification.**

All animal procedures were conducted by licensed professionals in accordance with the European Communities Directive 2010/63/EU and were approved by the Estonian National Board of Animal Experiments (permit no. 150, issued 27 September 2019). Lines 673-679 (methods, animals’ section)

**15. Housing and husbandry**

**Provide details of housing and husbandry conditions, including any environmental enrichment.**

Methods, animals’ section.

As enrichment items, nest-building materials and wooden blocks were provided.

**16. Animal care and monitoring**

1. **Describe any interventions or steps taken in the experimental protocols to reduce pain, suffering and distress.**

Methods, animals’ section, paragraph 1.

All animals were monitored regularly for signs of illness, injury, or trauma, including potential injuries related to fighting. No additional procedures were performed that would be expected to cause pain, distress, or injury; therefore, no specific analgesic or other interventions were required.

1. **Report any expected or unexpected adverse events.**

No adverse events took place during the animal experiments.

1. **Describe the humane endpoints established for the study, the signs that were monitored and the frequency of monitoring. If the study did not have humane endpoints, state this.**

During the behavioural experiments period, all animals were monitored daily for signs of weight loss and injuries that could potentially be caused by group housing. After the behavioural experiments, mice were euthanised by rapid decapitation using surgical scissors as the primary method, allowing the collection of both trunk blood and brain tissues. No anaesthesia was used as it could confound the interpretation of downstream molecular analyses, including qPCR and mass spectrometry.

**17. Interpretation/scientific implications**

1. **Interpret the results, taking into account the study objectives and hypotheses, current theory and other relevant studies in the literature.**

Discussion in the manuscript.

1. **Comment on the study limitations, including potential sources of bias, limitations of the animal model, and imprecision associated with the results.**

Discussion in the manuscript, limitations section.

**18. Generalisability/translation**

**Comment on whether, and how, the findings of this study are likely to generalise to other species or experimental conditions, including any relevance to human biology (where appropriate).**

**Ans:** The *Negr1^-/-^* mouse model has translational relevance, as NEGR1 is associated with neuropsychiatric and cognitive traits in humans. The behavioural and molecular findings observed here may therefore reflect mechanisms relevant to human neuropsychiatric disorders. However, extrapolation to humans should be made cautiously, as species-specific differences and the controlled experimental context may limit generalisability.

**19. Protocol registration**

**Provide a statement indicating whether a protocol (including the research**

**question, key design features, and analysis plan) was prepared before the study, and if and where this protocol was registered.**

All animal procedures were conducted in accordance with the European Communities Directive 2010/63/EU and were approved by the Estonian National Board of Animal Experiments (permit no. 150, issued 27 September 2019). The approved application included the research question and key elements of the study design. No separate preregistered protocol was filed.

**20. Data access**

**Provide a statement describing if and where study data are available.**

Data availability in the manuscript.

**21. Declaration of interests**

1. **Declare any potential conflicts of interest, including financial and non-financial. If none exist, this should be stated.**

Conflict of interest statement in the manuscript.

1. **List all funding sources (including grant identifier) and the role of the funder(s) in the design, analysis and reporting of the study.**

Funding in the manuscript.

The research was conducted using the research infrastructure “National Centre for Translational and Clinical Research”, funded by the Estonian Research Council (TARISTU24-TK22)

The funding provided a place to perform experiments, such as an animal facility and laboratory space, as well as reagents needed for qPCR and mass spectrometry, and software subscriptions for data analysis and visualisation tools like BioRender and GraphPad.
